# Upregulation of sperm-associated antigen 5 expression in endometrial carcinoma was associated with poor prognosis and immune dysregulation, and promoted cell migration and invasion

**DOI:** 10.1038/s41598-024-64354-4

**Published:** 2024-06-11

**Authors:** Manru Chen, Dan Wang, Yanyu Xu, Chenggang Yang

**Affiliations:** 1 Department of Obstetrics and Gynecology, Sichuan Academy of Medical Sciences & Sichuan Provincial People‘s Hospital, Chengdu, China; 2 Department of BigData, Beijing Medintell Bioinformatic Technology Co., LTD, Beijing, China; 3 Department of Research and Development, Gu’an Bojian Bio-Technology Co., LTD, Langfang, China

**Keywords:** Endometrial carcinoma, SPAG5, Functional enrichment, Migration, Invasion, Immune, Cancer, Diseases, Pathogenesis

## Abstract

Sperm-associated antigen 5 (SPAG5) regulates cancer cell invasion and is involved in the progression of many cancers. However, the role of SPAG5 in endometrial carcinoma (EC) is still unknown. The purpose of this study was to explore the role of SPAG5 in EC and its potential molecular mechanism. The UALCAN tool and cBioPortal were used to analyze the expression and alterations of SPAG5 in EC, respectively. OncoLnc was used for survival analysis. We analyzed the effects of SPAG5 on immune cell infiltration and the expression levels of immune checkpoints. We also overexpressed and knocked down SPAG5 in EC cells to explore the effect of SPAG5 regulation on migration, invasion, apoptosis, and the cell cycle of EC cells. We found that SPAG5 was overexpressed and the SPAG5 gene was often mutated in EC. High SPAG5 expression was significantly associated with poor overall survival in patients with EC. SPAG5 also affected the level of immune cell infiltration in the TIME and the expression of immune checkpoints lymphocyte activating 3 (LAG3) and T cell immunoreceptor with Ig and ITIM domains (TIGIT) in patients with EC. It may also be involved in the immunotherapy response in these patients. In vitro experiments showed that SPAG5 promotes cancer cell migration and invasion. In conclusion, this study lays the foundation for further understanding the molecular mechanisms of EC involving SPAG5 and contributes to diagnosing and managing this disease.

## Introduction

Endometrial carcinoma (EC, also known as UCEC) is a common gynecologic cancer. In common, status of endometrial cells are strongly affected by menstrual cycle^1^. EC is usually diagnosed in perimenopausal and postmenopausal women^2^. Up to 20% of patients with EC are at risk of recurrence and death^3^. The incidence of EC has increased recently. Surgery remains the primary therapeutic option for EC. However, radiotherapy, chemotherapy, and hormonal drugs are commonly used to treat patients with advanced or recurrent EC^4,5^. Despite advances in treatment in recent years, the 5-year survival rate of metastatic EC remains low, and the recurrence rate remains high^6^. Molecular diagnosis is a part of modern personalized medical diagnosis and treatment strategy^7^. Moreover, the diversity of molecular alterations may be a fundamental cause of the heterogeneity in the prognosis of EC^8,9^. Therefore, efforts have been made to identify more accurate molecular markers that complement currently known prognostic factors^10,11^.

Sperm-associated antigen 5 (SPAG5) is a mitotic spindleassociated protein^12–14^, that plays an important role in cell division^15,16^. Studies have found that SPAG5 is overexpressed in many human cancers and acts as an oncogene. The high expression of SPAG5 in gastric cancer is associated with poor prognosis, promoting the progression of gastric cancer by promoting the Wnt/β-catenin/survivin axis^17^. SPAG5 also promotes the invasion of breast cancer cells by activating Wnt/β-catenin signaling^18^. SPAG5 participates in human gliomagenesis by regulating cell proliferation and apoptosis ^19^. SPAG5 upregulation predicts poor prognosis in patients with cervical cancer and alters sensitivity to taxol therapy via the mTOR signaling pathway ^20^. SPAG5 is regulated by splicing factor 3b subunit 4 (SF3B4) and is involved in the migration of cervical cancer ^21^. Previous studies found that SPAG5 was associated with prognosis and immune cell infiltration in hepatocellular carcinoma and lung adenocarcinoma^22,23^. However, the role of SPAG5 in EC immunomodulation and disease progression and its molecular mechanisms are unknown. The purpose of this study was to explore the role of SPAG5 in EC and its potential molecular mechanism.

A large amount of data based on public databases is helpful for the identification of EC molecular markers^24^. In the current study, we compared the transcript levels of SPAG5 in EC and adjacent tissues. Subsequently, the effects of SPAG5 expression on immune cell infiltration, immune checkpoint expression, and immunotherapy responses were analyzed. In addition, we also overexpressed and knocked down the expression of SPAG5 in EC cells to explore its effects on migration, invasion, apoptosis, and the cell cycle in EC cells. Based on the results of this study, it is speculated that the upregulation of SPAG5 expression in EC is associated with poor prognosis and immune dysfunction, as well as promoting cell migration and invasion.

## Materials and Methods

### Bioinformatics analyses of SPAG5 gene expression and alterations

The UALCAN^25^ (http://ualcan.path.uab.edu/analysis.html) was used to analyze SPAG5 mRNA expression in EC samples from The Cancer Genome Atlas^26^ (TCGA). The Human Protein Atlas database^27^ (https://www.proteinatlas.org/) was used to analyze SPAG5 protein expression in normal and EC tissues. The cBioPortal^28^ (http://cbioportal.org) was used to analyze SPAG5 alterations in EC samples from TCGA. The search parameters included mutations, copy number variations (CNVs), and mRNA expression. OncoLnc^29^ (www.oncolnc.org/) was used for survival analysis. A Kaplan–Meier plotter (kmplot.com/) was used to evaluate the correlation between SPAG5 expression and the prognosis of patients with EC.

### Identification of genes related to or similar to SPAG5

GeneMANIA^30^ (http://www.genemania.org) was used to construct a network of genes that interact with SPAG5 and to predict the functions of these genes. In addition, the regulatory relationships among these interacting genes were analyzed based on STRING database^31^ (https://cn.string-db.org/). GEPIA^32^ (http://gepia.cancer-pku.cn/) was used to identify genes similar to SPAG5. Genes similar to SPAG5 were obtained using the Pearson correlation coefficient method, and those with r ≥ 0.5 were selected. These SPAG5-similar genes were then subjected to gene ontology (GO) and Kyoto Encyclopedia of Genes and Genomes (KEGG) functional enrichment analysis using GeneCoDis3^33^ (http://genecodis.genyo.es/?tdsourcetag=s_pcqq_aiomsg). KEGG contains numerous signaling pathways^34–36^. The screening criteria was set at *p*-value (*P*) < 0.05.

The LinkedOmics database^37^ (http://www.linkedomics.org/login.php) was used to identify which genes were co-expressed with SPAG5. The screening criteria were *P* < 0.05 and |r|≥ 0.5. Based on the existing protein interaction data from the BioGRID database^38^ (https://thebiogrid.org), Cytoscape software (version 3.6.1) was used to search for SPAG5 co-expressed genes. Non-co-expressed genes were removed to map protein network interactions. The SPAG5 co-expressed genes were then subjected to GO and KEGG functional enrichment analysis using GeneCoDis3.

### Immunological correlation analysis of SPAG5

The gene set marking each immune cell type infiltrating the tumor immune microenvironment (TIME) was obtained from Charoentong’s study^39^. The scores calculated using single sample gene set enrichment analysis (ssGSEA) were used to represent the relative abundance of immune cell infiltration in each sample TIME. Based on the median expression of SPAG5, patients with EC were divided into high and low expression groups, and the differences in immune cell infiltration between the two groups were compared. The immune, stromal, and ESTIMATE scores and the tumor purity of each patient with EC were also calculated using the ESTIMATE algorithm. The Wilcoxon test was used to analyze significant differences in immune cell infiltration, the immune, stromal, and ESTIMATE scores, and tumor purity between the two groups. Pearson’s correlation analysis was used to analyze the correlation between SPAG5 and differentially infiltrated immune cells. Subsequently, the TIMER database^40^ (https://cistrome.shinyapps.io/timer/) was used to analyze the influence of SPAG5 somatic mutations on immune cell infiltration. The expression of common immune checkpoints CD274 molecule (CD274), cytotoxic T-lymphocyte associated protein 4 (CTLA4), lymphocyte activating 3 (LAG3), programmed cell death 1 (PDCD1) and T cell immunoreceptor with Ig and ITIM domains (TIGIT) in the high and low expression groups was also analyzed.

To explore the effects of SPAG5 on immunotherapy, data were obtained from the GSE61676 dataset, which was annotate according to GPL5188. The 0-h survival samples were removed, and the remaining 43 samples were divided into high and low expression groups based on their median SPAG5 expression. Subsequently, the proportions of immunotherapy-responsive and non-responsive patients in the high and low expression groups were analyzed, and the expression of SPAG5 was compared between the immunotherapy-responsive and non-responsive groups. In addition, Kaplan Meier analysis was used to analyze the survival differences between the high and low expression groups.

### Identification of SPAG5 methylation sites and construction of SPAG5-miRNA network

Epigenetic modification plays an important regulatory role in human diseases^41,42^. In this study, methylation sites of the SPAG5 gene were identified based on the Wanderer database^43^ (http://maplab.imppc.org/wanderer). Adj.*p*val < 0.05, implying that methylation site was significantly different in the control and cancer groups. In addition, miRNAs targeting SPAG5 were identified based on the ENCORI database^44^ (http://starbase.sysu.edu.cn/index.php), and the SPAG5-miRNA network was constructed.

### Quantitative real-time PCR (qRT-PCR) validation in tissue samples

Nine patients with EC were enrolled in this study. The inclusion criteria were as follows: (1) Patients were first diagnosed with EC according to the diagnostic guidelines for EC and confirmed by pathological histologic examination; (2) Patients had not undergone radiotherapy or chemotherapy prior to surgery; (3) Patients had complete clinical information, including age, blood pressure, family history, etc.. Patients with other malignant tumors or viral infection, incomplete clinical information, and recurrence were excluded. EC tissue samples (cancerous tissue) and para-carcinoma tissue samples (normal control group) were collected for qRT-PCR. The qRT-PCR has been used extensively to analyze gene expression^45,46^. Total RNA was extracted using TRIzol (10,296,028, Invitrogen, California, USA). Reverse transcription was performed using FastKing cDNA first strand synthesis kit (KR116, TIANGEN, Beijing, China). QRT-PCR was performed using SuperReal PreMix Plus (SYBR Green) (FP205, TIANGEN, Beijing, China). Glyceraldehyde-3-phosphate dehydrogenase (GAPDH) and actin beta (ACTB) were internal parameters for qRT-PCR standardization. The experiment was independently repeated three times for each sample. Relative gene expression levels were calculated using the 2 ^−ΔΔCt^ method^47^.

### Knockdown and overexpression of SPAG5 in cells

QRT-PCR was used to detect the expression of SPAG5 in human endometrial stromal cells (hESCs), HEC-1-A, AN3CA, Ishikawa, and RL-95-2 cells to screen suitable cells for subsequent experiments. Cells were then selected for SPAG5 overexpression and knockdown. Subsequently, the expression levels of SPAG5 in overexpression and knockdown cells were detected using qRT-PCR. Western blotting was then performed to detect SPAG5 protein expression. Proteins were extracted from cells in the logarithmic growth phase using a protein extract (MDL91201, MDL, Beijing, China). The concentration of the extracted protein was determined using BCA protein concentration determination kit (MD913053, MDL, Beijing, China). Protein bands were separated using sodium dodecyl sulfate polyacrylamide gel electrophoresis prefabricated adhesive kit (MD911919, MDL, Beijing, China). Then, the protein bands separated on the gel were transferred to polyvinylidene fluoride (PVDF) membrane by transfer electrophoresis (ISEQ00010, Millipore, Massachusetts, USA). The PVDF membrane was incubated with the primary antibody (SPAG5 antibody, YN1358, ImmunoWay, Texas, USA, diluted 1:2000) at 4 °C overnight, and then incubated with the corresponding secondary antibody at room temperature for 60 min.

### *Detection of the cell cycle *via* flow cytometry*

Flow cytometry was used to detect the effects of SPAG5 on the cell cycle. Cell sample suspension was prepared, and 1 ml was then added dropwise to 4 ml of 95% ethanol (pre-cooled in an ice bath) and vortexed at low speed. After mixing, the cells were fixed at 4 °C for at least 2 h. The cells were centrifuged for 3–5 min (1,000 rpm/min) to remove the supernatant. The cells were re-suspended by adding 5 ml pre-cooled phosphate buffer saline (PBS) (MD911702, MDL, Beijing, China) in ice bath and centrifuged again to remove supernatant. Then, 0.1 ml RNaseA solution was added to the cell precipitate, the cells were re-suspended, and incubated at 37 °C for 30 min. Afterward, 0.4 ml of propidium iodide (PI) staining solution was added to the cells and were mixed well. Finally, the cell suspensions were incubated at 37 °C for 30 min in the dark for flow cytometry detection.

### *Detection of cell apoptosis *via* flow cytometry*

Flow cytometry was also used to determine the effects of SPAG5 on cell apoptosis. The cells were detached using trypsin free of ethylene diamine tetraacetic acid (EDTA), then cell culture solution was added, and the cells were gently dispersed. Cells were collected via centrifugation for 5 min (1000 rpm/min), and the supernatant was removed. Then, the cells were re-suspended with 1 ml PBS (pre-cooled at 4 °C), centrifuged again, and the supernatant was discarded. The cells were re-suspended with 1X binding buffer and the cell density was adjusted to 1*10^6^ cells/ml. Then, 5 μl Annexin V-FITC and 100 μl of the cell suspension were mixed in a 5 ml flow tube and dark incubated at room temperature for 5 min. Next, 10 μl PI solution and 400 μl PBS were added and flow cytometry was performed immediately.

### Cell wound scratch and Transwell assays

Cell wound scratch assay was performed to analyze the effect of SPAG5 on cell migration. Scratches were made using micro pipette tip, and the scratched cells were removed by washing with PBS for 3 times. Then, serum-free medium was added, and the cells were placed in 5% carbon dioxide incubator at 37˚C for culture. After that, the samples were taken according to the culture time of 0 h, 24 h, and 48 h and photographed.

Transwell assay was also used to detect the effect of SPAG5 on cell migration and invasion. All cell culture reagents and Transwell chambers were incubated at 37˚C. First, cells were re-suspended in serum-free medium at a density of 2*10^5^ cell/ml. Next, 700 µl of the culture medium containing 10% serum was added to the lower chamber, 150 µl of the cell suspension was added to the upper chamber, and the cells were cultured in an incubator for 24 h. After incubation, the chamber was carefully removed using tweezers, the excess liquid in the upper chamber was blotted out, and the Transwell was transferred to a well pre-filled with approximately 800 µl of methanol, fixing at room temperature for 30 min. Next, the chamber was removed, excess fixative solution was blotted from the upper chamber, and was transferred to a well pre-filled with approximately 800 µl Giemsa dye. The chamber was stained at room temperature for 15–30 min. Gently rinse and soak in water for several times, remove the chamber, absorb the liquid from the upper chamber, and carefully wipe off the cells on the surface of the membrane at the bottom of the upper chamber with a wet cotton swab. The membrane was carefully removed using small tweezers, dried with the bottom side up, and transferred to a glass slide. Finally, the membrane was sealed with neutral gum and observed under a microscope.

In addition, the steps for the Transwell cell invasion analysis are as follows. First, Matrigel was thawed at 4 °C overnight and diluted with serum-free medium pre-cooled at 4 °C to a final concentration of 1 mg/ml, on ice. Next, 100 µl of the diluted Matrigel was added vertically in the center of the bottom of the upper chamber of the Transwell and incubated at 37 °C for 4–5 h to dry into a gel. The steps for cell incubation and chamber preparation for analysis were performed as in the migration assay above.

### Statistical analysis

All experiments were repeated independently at least three times. Herein, data are presented as the mean ± standard deviation. GraphPad Prism software (Version 8.0.2, GraphPad, Massachusetts, USA) was used to perform all statistical analyses. The T-test and one‑way ANOVA were used for significance analysis of two and more groups, respectively. *P* < 0.05 was considered statistically significant. The Shapiro–wilk test function was used to test the normality distribution of data. Levene’s test was used to test for homogeneity of variance. The results of Levene’s test showed homogeneity of variance, and the LSD method was used for post hoc test. The results of Levene’s test showed heterogeneity of variance, and the Games-Howell method was used for post hoc test.

### Ethical approval

Approval of the research protocol by an Institutional Reviewer Board: This study was approved by the ethics committee the Sichuan Provincial People’s Hospital (No.2022213). All participants were informed as to the purpose of this study, and that this study complied with the Declaration of Helsinki. The informed consent was obtained from the all participants.

## Results

### SPAG5 expression is upregulated in EC

**Figure 1:**
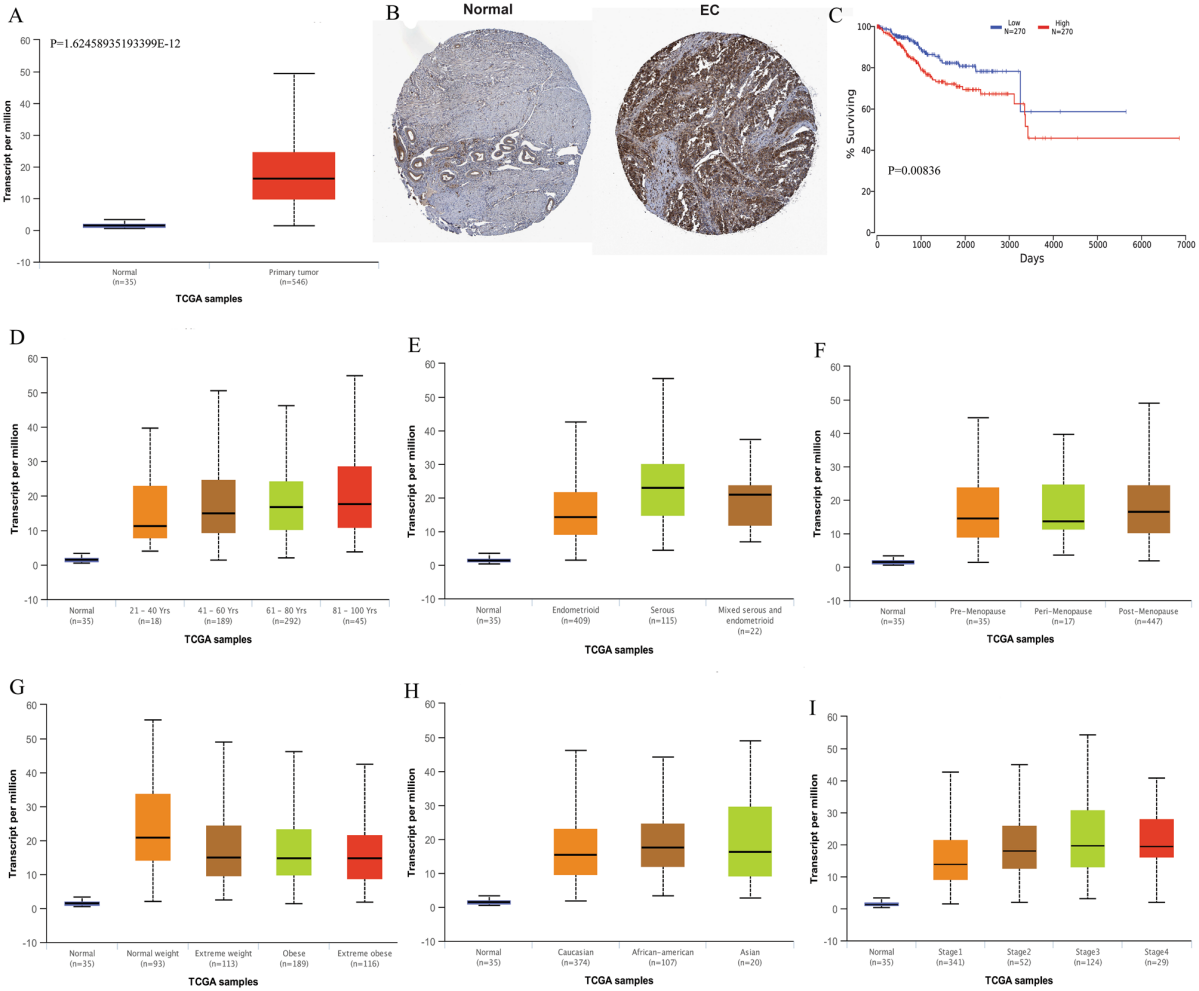
Expression and prognostic analysis of SPAG5. (**A**), The expression of SPAG5 mRNA in EC tissues and adjacent tissues in TCGA database; (**B**), The expression of SPAG5 protein in normal tissues and EC tissues in the Human Protein Atlas; (**C**), Effect of SPAG5 expression on survival of EC patients in TCGA database; (**D**), Expression of SPAG5 in different ages of EC patients; (**E)**: Expression of SPAG5 in different histological subtypes of EC patients; (**F**), Expression of SPAG5 in different menopause status of EC patients; (**G)**, Expression of SPAG5 in different weight of EC patients; (**H**), Expression of SPAG5 in different race of EC patients; (**I**), Expression of SPAG5 in different stages of EC patients. TCGA, the cancer genome atlas; EC, endometrial carcinoma.

Differential expression analysis showed that SPAG5 mRNA expression level was significantly higher in EC tissues than in normal controls (*P *˂ 0.01, Fig. 1A). Moreover, SPAG5 protein expression level was also significantly increased in EC tissues than in normal controls (Fig. 1B). OncoLnc was used to evaluate the prognostic value of SPAG5 in TCGA. As shown in Fig. 1C, high mRNA expression of SPAG5 (*P* = 0.00836) was significantly associated with poor overall survival (OS) of patients with EC. This suggests that high SPAG5 expression is associated with poor prognosis of EC and may be used as a biomarker to predict the survival of patients with EC. As shown in Fig. 1D–I, the SPAG5 was significantly higher in patients with EC than healthy people in subgroup analyses based on age, weight, race, menopause status, histological subtypes and cancer stages. The statistical significance was shown in Table S1.

### Frequency and type of SPAG5 alterations in EC

The cBioPortal was used to analyze the types and frequencies of SPAG5 alterations in EC. SPAG5 was altered in 79 of 527 (15%) patients with EC (Fig. S1A and B). These alterations were mRNA high in 31 cases (5.88%), amplification in 3 cases (0.57%), mutation in 31 cases (5.88%), and multiple alterations in 14 cases (2.66%). Thus, mutations are the most common type of SPAG5 CNV in EC. The expression level of SPAG5 in different altered states is shown in Fig. S1C.

### Genes interacting with SPAG5

**Figure 2:**
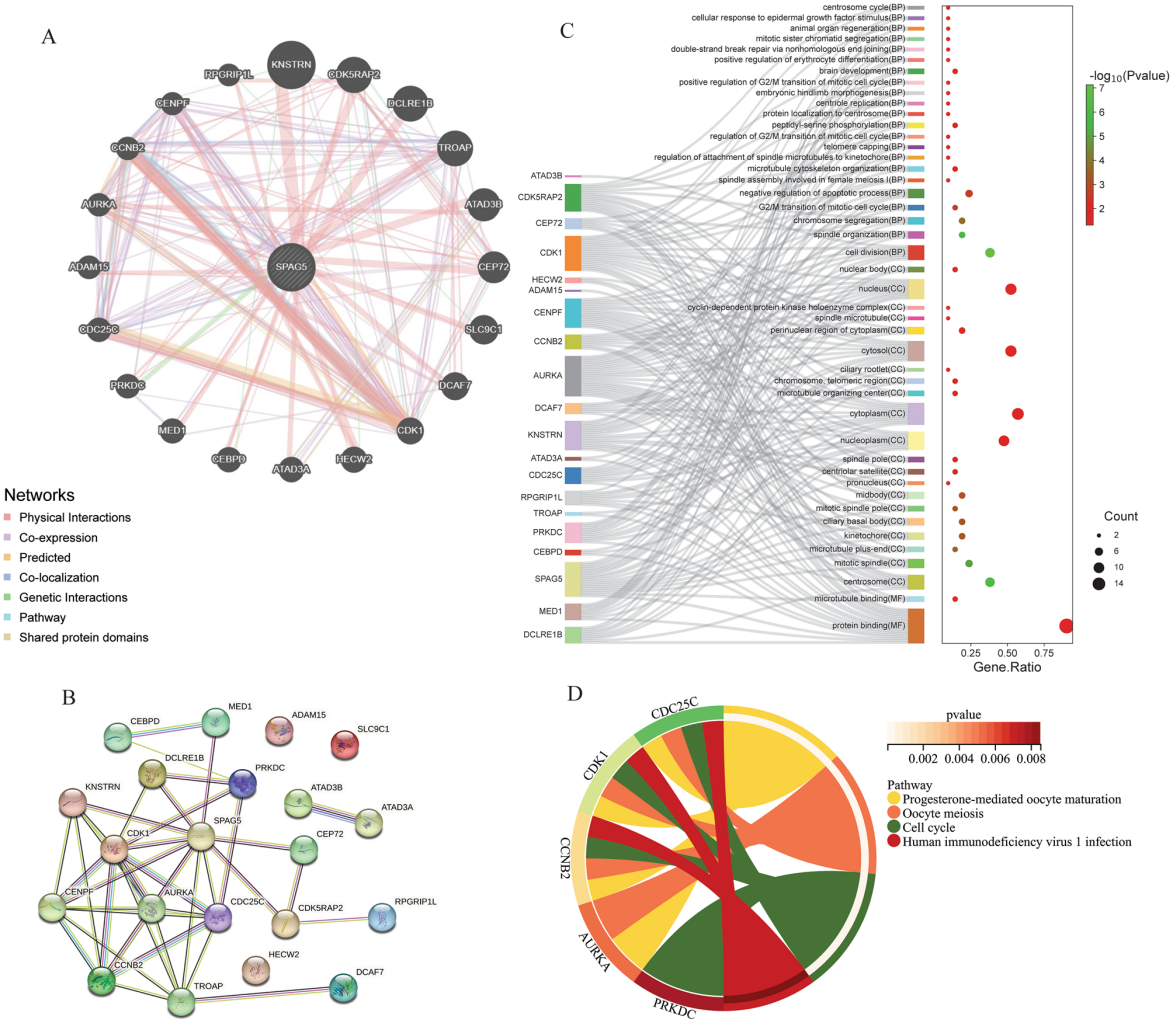
PPI network and functional enrichment of genes interacting with SPAG5. (**A**), PPI network of genes interacting with SPAG5 based on GeneMANIA database; (**B**), PPI network of genes interacting with SPAG5 based on STRING database; (**C**), GO functional enrichment of genes involved in the PPI network; (**D**), KEGG functional enrichment of genes involved in the PPI network.

The protein–protein interaction (PPI) network analysis of the genes interacting with SPAG5 was performed using GeneMANIA to obtain the interaction relationship between the genes and the main functions involved. As shown in Fig. 2A, the genes that interacted with SPAG5 included kinetochore localized astrin (SPAG5) binding protein (KNSTRN), CDK5 regulatory subunit associated protein 2 (CDK5RAP2), DNA cross-link repair 1B (DCLRE1B), trophinin associated protein (TROAP), ATPase family AAA domain containing 3B (ATAD3B), centrosomal protein 72 (CEP72), solute carrier family 9 member C1 (SLC9C1), DDB1 and CUL4 associated factor 7 (DCAF7), cyclin dependent kinase 1 (CDK1), HECT, C2 and WW domain containing E3 ubiquitin protein ligase 2 (HECW2), ATPase family AAA domain containing 3A (ATAD3A), CCAAT enhancer binding protein delta (CEBPD), mediator complex subunit 1 (MED1), protein kinase, DNA-activated, catalytic subunit (PRKDC), cell division cycle 25C (CDC25C), ADAM metallopeptidase domain 15 (ADAM15), aurora kinase A (AURKA), cyclin B2 (CCNB2), centromere protein F (CENPF), and RPGRIP1 like (RPGRIP1L). This network contains 21 nodes and 279 edges and does not have directionality. In addition, results based on the STRING database showed that SPAG5 had the highest interactions scores with CDK1 (score = 0.927), CENPF (score = 0.905) and KNSTRN (score = 0.983) (Fig. 2B and Table S2).

GO and KEGG analyses were performed on the node genes in the PPI network (Fig. 2C, D and Table S3). A total of 22 biological process (BP) terms, 21 cellular component (CC) terms and 2 molecular function (MF) terms were obtained by GO enrichment. Moreover, it was found that SPAG5 was enriched in 19 terms such as ciliary basal body(CC), cell division(BP); spindle organization(BP); chromosome segregation(BP); regulation of attachment of spindle microtubules to kinetochore(BP). Four significantly enriched signaling pathways were identified by KEGG analysis, namely progesterone-mediated oocyte maturation, oocyte meiosis, cell cycle and human immunodeficiency virus 1 infection. The results showed that these genes are involve in multiple biological pathways, which helps to understand the regulatory function of the SPAG5.

### GO and KEGG pathway analyses of genes similar to SPAG5 in EC

Using GEPIA, genes similar to SPAG5 in EC tissues were obtained using the Pearson correlation coefficient method and are displayed in Table S3. The results showed that SPAG5 was most similar to TROAP. Significant GO term analysis using GeneCoDis3 showed that genes similar to SPAG5 were mainly enriched in cell division, mitotic cell cycle, cell population proliferation, protein binding, ATP binding and nucleus. KEGG pathway analysis revealed enrichment in the cell cycle, oocyte meiosis and progesterone-mediated oocyte maturation pathways (Fig. S2).

### Functional enrichment analyses of co-expression genes correlated with SPAG5 in EC

As shown in the volcano plot (Fig. S3), 409 genes (dark red dots) were positive correlations with SPAG5, whereas 22 genes (dark green dots) were negative correlations (*P* < 0.05, |r|≥ 0.5). In the PPI network, 345 nodes and 1125 edges illustrated the interactions between the co-expressed genes of SPAG5. Among which, exportin 1 (XPO1), cyclin dependent kinase 2 (p33, also known as CDK2), BRCA1 DNA repair associated (RNF53), minichromosome maintenance complex component 2 (MITOTIN) and HECT, UBA and WWE domain containing E3 ubiquitin protein ligase 1 (UREB1) were hub proteins which have high degree (Fig. S4A). Significant GO term analysis using GeneCoDis3 showed that genes differentially expressed in correlation with SPAG5 were mainly involved in the cell division, DNA replication, protein binding and RNA binding. KEGG pathway analysis showed enrichment in the cell cycle, oocyte meiosis, DNA replication and progesterone-mediated oocyte maturation pathways. The top15 GO and KEGG items were shown in Fig. S4B–E.

### Immunological correlation analysis of SPAG5

**Figure 3:**
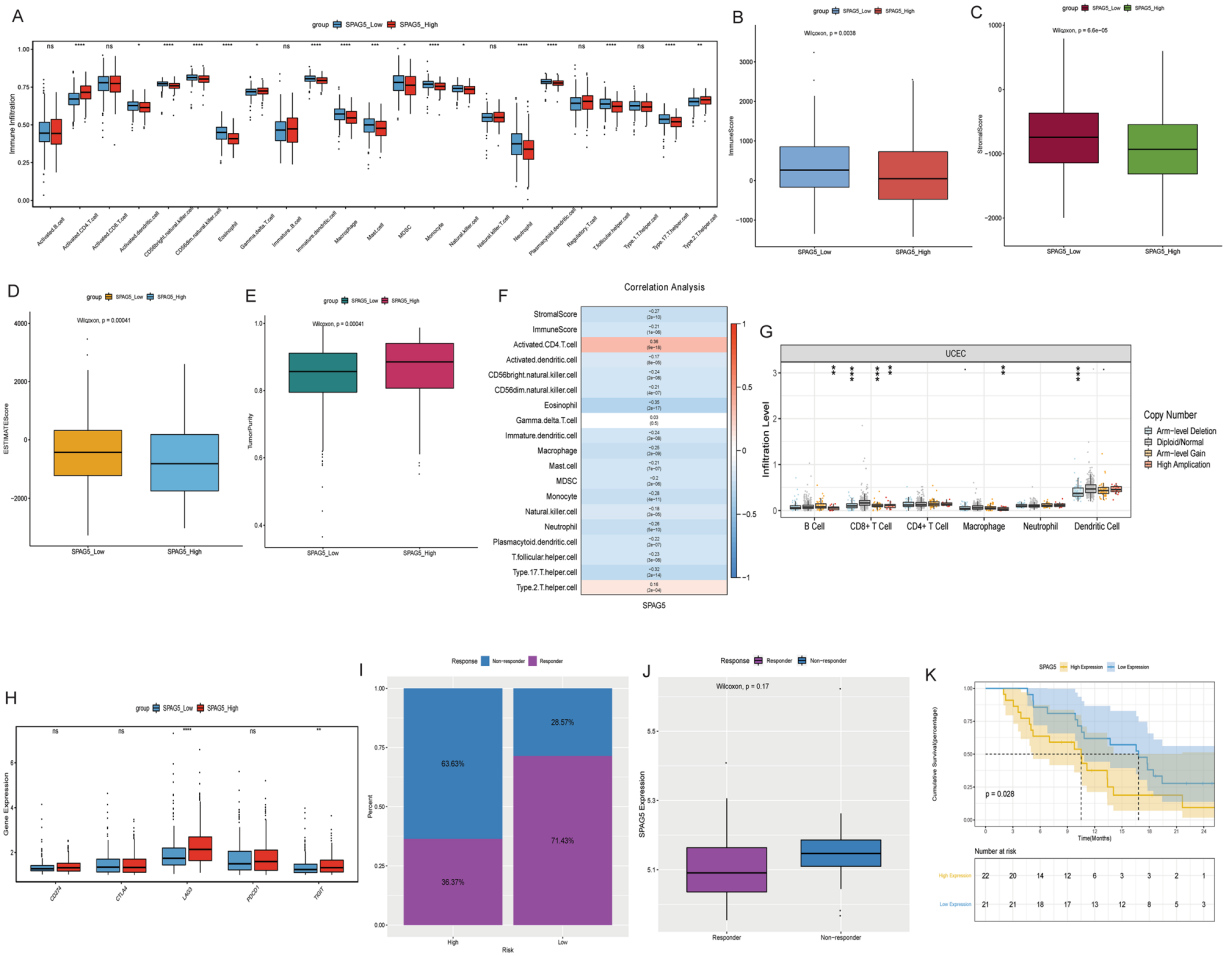
Immunological correlation analysis of SPAG5. **(A**), Analysis of the difference of immune cell infiltration degree between high and low expression groups of SPAG5; (**B**), Analysis of the difference of immune score between high and low expression groups of SPAG5; (**C**), Analysis of the difference of stromal score between high and low expression groups of SPAG5; (**D**), Analysis of the difference of ESTIMATE score between high and low expression groups of SPAG5; (**E**), Analysis of the difference of tumor purity between high and low expression groups of SPAG5; (**F**), Pearson correlation between SPAG5 and differential infiltrated immune cells, immune score, and stromal score; (**G**), The influence of SPAG5 somatic mutation on immune cell infiltration was analyzed based on TIMER database; (**H**), Differential expression analysis of common immune checkpoints CD274, CTLA4, LAG3, PDCD1 and TIGIT in high and low expression groups of SPAG5; (**I**), Analysis of the proportion of immunotherapy responsive patients and non-responsive patients in high and low expression groups of SPAG5 based on GSE61676 dataset; (**J**), The difference of SPAG5 expression between the immunotherapy-responding group and the non-responding group based on GSE61676 dataset; (**K**), Kaplan Meier analyzed survival differences between high and low expression groups of SPAG5 based on GSE61676 dataset.

SSGSEA analysis showed that the infiltration degree of most immune cells, such as Activated.dendritic.cell, Macrophage and Neutrophil, in the low expression group was significantly higher than that in the high expression group (Fig. 3A). ESTIMATE analysis showed that the low expression group had higher immune score, stromal score, ESTIMATE score than the high expression group, and also had lower tumor purity (Fig. 3B–E). Pearson correlation analysis showed that SPAG5 was negatively correlated with most of the differential infiltrated immune cells (Fig. 3F). Moreover, SPAG5 somatic mutations also effected immune cell infiltration; however, different mutation types may affect immune cell infiltration differently (Fig. 3G). We also found that SPAG5 might affect the expression of the immune checkpoints LAG3 and TIGIT (Fig. 3H). In addition, patients with low SPAG5 expression had more sensitive immune response and higher survival rate (Fig. 3I–K). These results suggest that SPAG5 may affect immune regulation and immunotherapy of patients with EC.

### SPAG5 methylation sites and SPAG5-miRNA network

Based on the Wanderer database, a total of 24 methylation sites of the SPAG5 gene were identified, of which 17 methylation sites were significantly different in the control and cancer groups (Fig. S5A). This result implies that methylation modification of SPAG5 may influence its regulatory role in EC. In addition, 18 miRNAs that may target SPAG5 were identified based on the ENCORI database (Fig. S5B). This again implies the complexity of the molecular mechanisms of SPAG5 in EC regulation.

### Expression validation of SPAG5 in vitro

**Figure 4:**
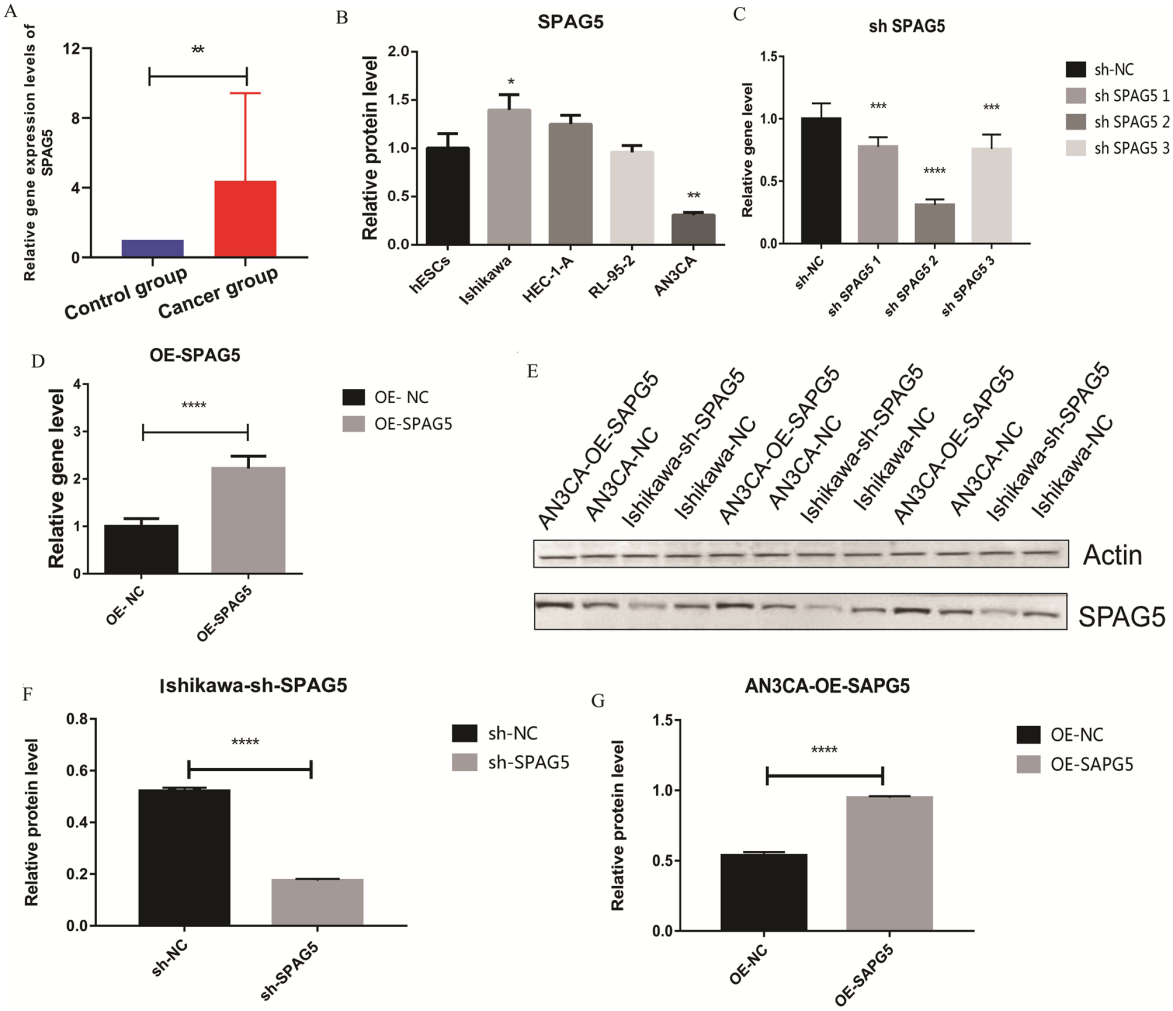
SPAG5 expression in clinical samples and overexpressed and knockdown EC cells. (**A**), QRT-PCR validation of SPAG5 in the tissue of patients with EC; (**B**), QRT-PCR was used to detect the expression of SPAG5 in hESCs, HEC-1-A, AN3CA, Ishikawa and RL-95–2 cells; (**C**), QRT-PCR was used to detect the expression of SPAG5 to screen out effective interference targets in Ishikawa cell; (**D**), qRT-PCR was used to detect the overexpression effect of SPAG5 in AN3CA cell; (**E**), The expression bands of SPAG5 protein in Ishikawa-NC, Ishikawa-sh, AN3CA-NC and AN3CA-OE cells were detected by western blotting; (**F**), The relative expression levels of SPAG5 protein in Ishikawa-NC and Ishikawa-sh cells; (**G**), The relative expression levels of SPAG5 protein in AN3CA-NC and AN3CA-OE cells. QRT-PCR, Quantitative real-time PCR; EC, endometrial carcinoma; Ishikawa-NC, Ishikawa cell-normal control; Ishikawa-sh, Ishikawa cell with knockdown of SPAG5 expression; AN3CA-NC, AN3CA cell-normal control; AN3CA-OE, AN3CA cell with overexpression of SPAG5. *, *P* < 0.05; **, *P* < 0.01; ***, *P* < 0.001 and ****, *P* < 0.0001.

Nine patients with EC were enrolled in this study. Patient clinical information and complete primers are shown in Tables S5 and S6, respectively. GAPDH and ACTB were internal parameters for qRT-PCR standardization. The qRE-PCR results showed that SPAG5 expression was higher in EC tissues than in normal tissues (Fig. 4A). Moreover, the expression of SPAG5 in hESCs, HEC-1-A, AN3CA, Ishikawa and RL-95-2 cells was detected via qRT-PCR. The results showed that compared with normal hESCs cell, the expression of SPAG5 in Ishikawa cell was significantly increased, while the expression of SPAG5 in AN3CA was significantly decreased (Fig. 4B). Therefore, Ishikawa (knockdown of SPAG5) and AN3CA (overexpression of SPAG5) were selected for later cell experiments.

Ishikawa cell was selected to knockdown the expression of SPAG5, and qRT-PCR was used to detect the expression of SPAG5 to screen out effective interference targets. The results showed that the knockdown effect of target 2 was the best (Fig. 4C), so we chose sh SPAG5 2 for subsequent experiments. AN3CA cell was selected to overexpress SPAG5, and qRT-PCR was used to detect the overexpression effect of SPAG5. The expression level of SPAG5 in AN3CA cell was significantly increased after overexpression (Fig. 4D). Subsequently, the expression of SPAG5 protein in the overexpression and knockdown cells was detected, and the results were consistent with the mRNA expression results (Fig. 4E–G). Full length uncropped blots in Supplementary material.

### SPAG5 promotes EC cells migration and invasion

**Figure 5:**
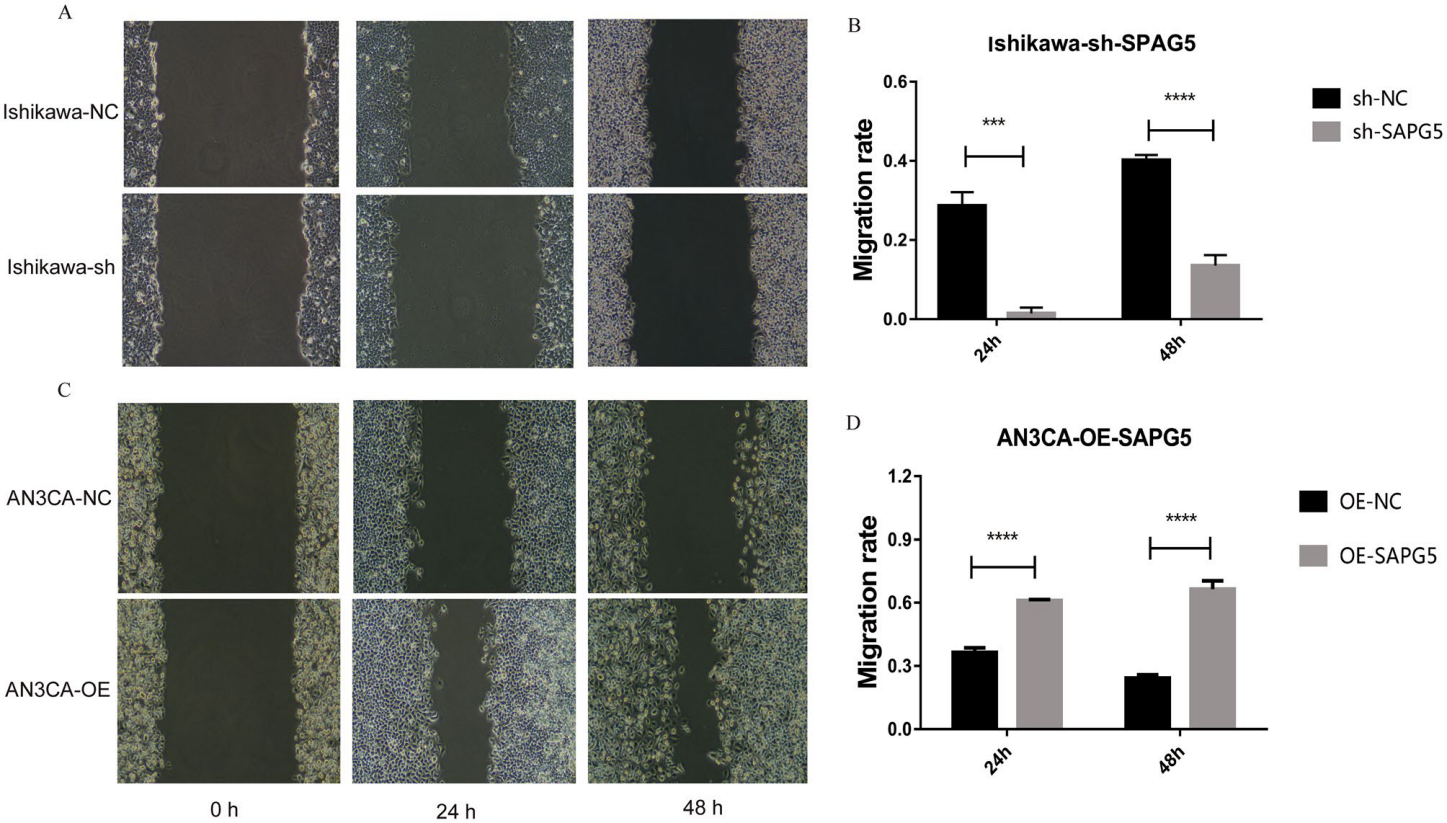
Migration of EC cells after overexpression and knockdown of SPAG5. (**A**), Migration ability of EC cells at 24 h and 48 h after knockdown of SPAG5; (**B**), Migration rate of Ishikawa-NC cell and Ishikawa-sh cell at different times; (**C**), Migration ability of EC cells at 24 h and 48 h after overexpression of SPAG5; (**D**), Migration rate of AN3CA-NC cell and AN3CA-OE cell at different times. EC, endometrial carcinoma; Ishikawa-NC, Ishikawa cell-normal control; Ishikawa-sh, Ishikawa cell with knockdown of SPAG5 expression; AN3CA-NC, AN3CA cell-normal control; AN3CA-OE, AN3CA cell with overexpression of SPAG5. *P* < 0.001 and ****, *P* < 0.0001.

**Figure 6:**
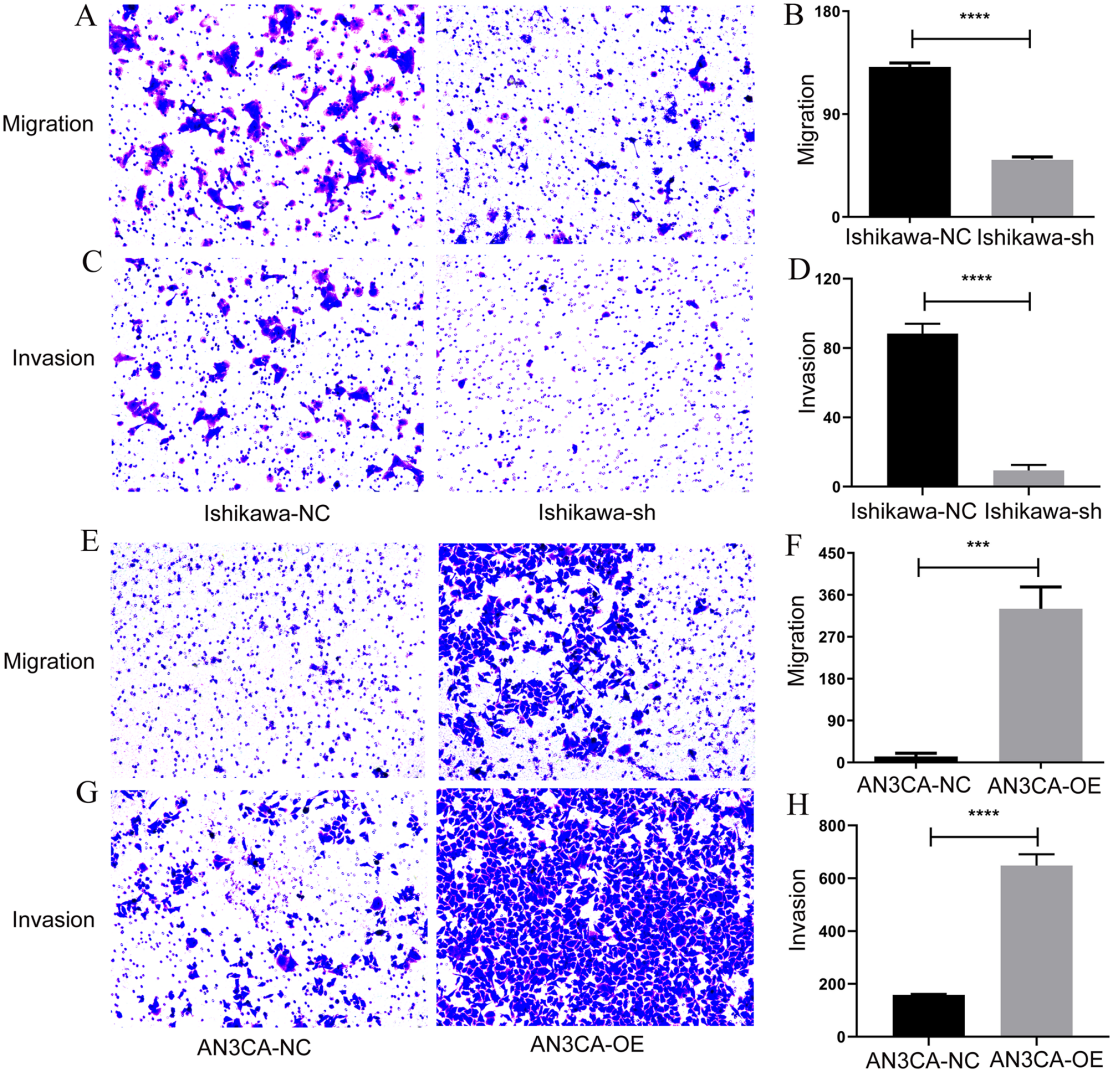
Transwell assay was used to detect the cell migration and invasion of EC after SPAG5 overexpression and knockdown. (**A**), Transwell assay was used to detect the cell migration of EC after SPAG5 knockdown; (**B**), Statistical analysis of Ishikawa cell migration after SPAG5 knockdown; (**C**), Transwell assay was used to detect the cell invasion of EC after SPAG5 knockdown; (**D**), Statistical analysis of Ishikawa cell invasion after SPAG5 knockdown; (**E**), Transwell assay was used to detect the cell migration of EC after SPAG5 overexpression; (**F**), Statistical analysis of AN3CA cell migration after SPAG5 overexpression; (**G**), Transwell assay was used to detect the cell invasion of EC after SPAG5 overexpression; (**H**), Statistical analysis of AN3CA cell invasion after SPAG5 overexpression. Ishikawa-NC, Ishikawa cell-normal control; Ishikawa-sh, Ishikawa cell with knockdown of SPAG5 expression; AN3CA-NC, AN3CA cell-normal control; AN3CA-OE, AN3CA cell with overexpression of SPAG5; EC, endometrial carcinoma. Observation with a 100 × microscope.

Cell wound scratch assay showed that the migration ability of Ishikawa cell was decreased at 24 h and 48 h after SPAG5 knockdown (Fig. 5A, B). Moreover, the migration ability of AN3CA cell was enhanced at 24 h and 48 h after SPAG5 overexpression (Fig. 5C, D). Transwell migration and invasion assays showed that the migration and invasion ability of Ishikawa cell was weakened after SPAG5 knockdown (Fig. 6A–D), while the migration and invasion ability of AN3CA cell was enhanced after SPAG5 overexpression (Fig. 6E–H).

### SPAG5 affects cancer cells cycle and apoptosis

**Figure 7:**
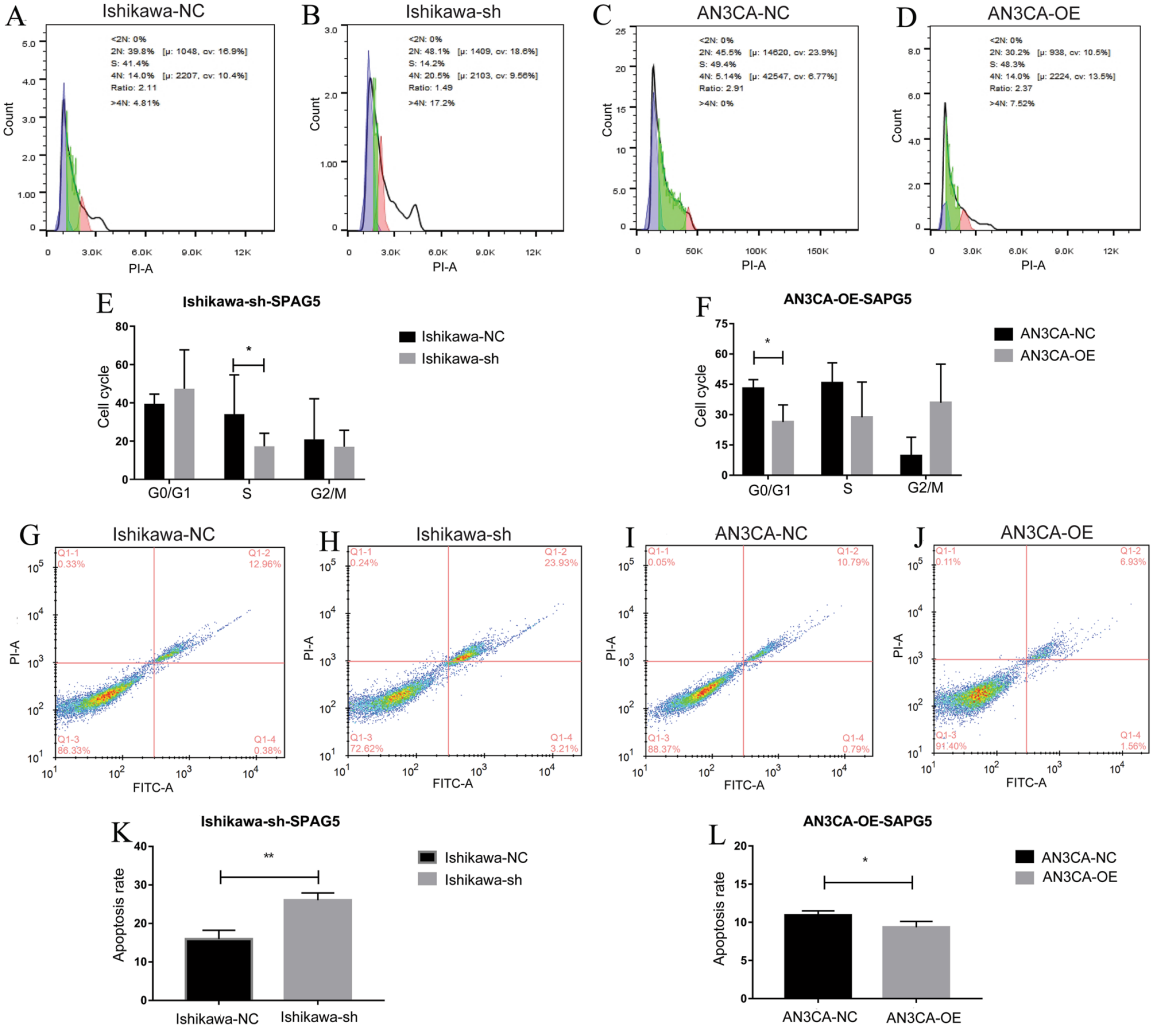
Flow cytometry was used to detect the cell cycle and apoptosis of EC after SPAG5 overexpression and knockdown. (**A**), Flow cytometry was used to detect normal Ishikawa cell cycle; (**B**), Flow cytometry was used to detect Ishikawa cell cycle after SPAG5 knockdown; (**C**), Flow cytometry was used to detect normal AN3CA cell cycle; (**D**), Flow cytometry was used to detect the AN3CA cell cycle after SPAG5 overexpression; (**E**), The flow cytometry cell cycle statistical histograms of Ishikawa-NC and Ishikawa-sh groups; (**F**), The flow cytometry cell cycle statistical histograms of AN3CA-NC and AN3CA-OE groups; (**G**), Flow cytometry was used to detect normal Ishikawa cell apoptosis; (**H**), Flow cytometry was used to detect Ishikawa cell apoptosis after SPAG5 knockdown; (**I**), Flow cytometry was used to detect normal AN3CA cell apoptosis; (**J**), Flow cytometry was used to detect the AN3CA cell apoptosis after SPAG5 overexpression; (**K**), The flow cytometry cell apoptosis statistical histograms of Ishikawa-NC and Ishikawa-sh groups; (**L**), The flow cytometry cell apoptosis statistical histograms of AN3CA-NC and AN3CA-OE groups. EC, endometrial carcinoma; Ishikawa-NC, Ishikawa cell-normal control; Ishikawa-sh, Ishikawa cell with knockdown of SPAG5 expression; AN3CA-NC, AN3CA cell-normal control; AN3CA-OE, AN3CA cell with overexpression of SPAG5.

After knockdown of SPAG5 in Ishikawa cell, the number of cells in G0/G1 phase increased, and the number of cells in S phase and G2/M phase decreased, indicating that the cells were blocked in G0/G1 phase after SPAG5 knockdown (Fig. 7A, B). After overexpression of SPAG5 in AN3CA cell, the number of cells in G0/G1 phase and S phase decreased, and the number of cells in G2/M phase increased, indicating that the cells were blocked in G2/M phase after SPAG5 overexpression (Fig. 7C, D). The corresponding flow cytometry cell cycle histograms are shown in Fig. 7E, F. In addition, the apoptotic capacity of the cells was enhanced after knockdown of SPAG5 in Ishikawa cell, whereas the apoptotic capacity of cells was decreased after overexpression of SPAG5 in AN3CA cells (Fig. 7G–J). This suggests that the expression of SPAG5 regulates the apoptotic ability of EC cells. The flow cytometry cell apoptosis statistical histograms are shown in Fig. 7K, L.

## Discussion

SPAG5 is a potential oncogene that plays a key role in the progression of a variety of tumors^48–50^. In this study, we found that SPAG5 expression is elevated in EC tissues. High SPAG5 expression of EC may be associated with poor prognosis. Moreover, the expression of SPAG5 may also affect the immunomodulatory mechanisms and response to immunotherapy in patients with EC. In addition, SPAG5 may regulate the migration, invasion, apoptosis, and cell cycle of EC cells, thus laying the foundation for the diagnosis and treatment of EC.

The upregulation of SPAG5 in cervical cancer is associated with poor prognosis^20^. Jue Jiang demonstrated that SPAG5 promotes the invasion of breast cancer cells by activating Wnt/β-catenin signaling via Wnt3 expression upregulation^51^. In this study, we found that SPAG5 was highly expressed in EC and was associated with poor prognosis. Furthermore, the transcription level of SPAG5 was related to EC patients’ age, weight, ethnicity, menopause status, pathological state and tumor grade. Thus, we speculated that SPAG5 expression may be a potential prognostic biomarker for EC. Moreover, the interaction between SPAG5 and KNSTRN was found to be the highest. KNSTRN is a hub gene in EC^52^. Therefore, it is speculated that KNSTRN may be involved in regulating the impact of SPAG5 on EC. In addition, it was found that SPAG5 was enriched in 19 terms such as ciliary basal body(CC), cell division(BP); spindle organization(BP); chromosome segregation(BP); regulation of attachment of spindle microtubules to kinetochore(BP). This result provides potential research directions for exploring the biological pathways involved in SPAG5. Using GEPIA, genes similar to SPAG5 in EC tissues were identified, among which TROAP was the most similar. TROAP was first identified as a participant in early embryo implantation^53,54^. TROAP also regulates spindle assembly and centrosome integrity during cell mitosis^55^. Previous studies have shown that TROAP is involved in cell proliferation and migration in many cancers^56–58^. TROAP is highly expressed in ovarian cancer, is associated with poor prognosis, and is considered a potential prognostic marker^56^. Therefore, exploring the mechanisms of action of TROAP and SPAG5 may contribute to our understanding of the molecular mechanisms of EC progression.

Immune regulation plays an important role in the progression of a variety of tumors, including EC^59,60^. In addition, the immune response is associated with molecular subtypes of EC^61^. Previous studies have shown that molecular abnormalities in EC are related to the prognosis of the disease and an imbalance of immune cell infiltration^62–64^. In addition, previous studies found that SPAG5 was associated with prognosis and immune cell infiltration in hepatocellular carcinoma and lung adenocarcinoma^22,23^. However, to date, no relevant studies have been conducted on the role of SPAG5 in EC immune regulation. In this study, ssGSEA analysis showed that the infiltration of most immune cells in the low expression group of SPAG5 was significantly higher than that in the high expression group of SPAG5. ESTIMATE analysis showed that the low expression group of SPAG5 had higher immune score, stromal score, ESTIMATE score than the high expression group of SPAG5, and also had lower tumor purity. Previous studies have shown that patients with cervical cancer with higher immune and ESTIMATE scores and lower tumor purity have a better prognosis^65–67^. Moreover, we found that SPAG5 may affect the expression of the immune checkpoints LAG3 and TIGIT. Positive expression of LAG3 in immune cells is more common in EC patients with high-risk characteristics^68^. The upregulated expression of TIGIT in cancer cells contributes to the local inhibition of immune surveillance, and blocking TIGIT signaling can improve the response of CD8^+^ T and natural killer cells^69^. In addition, we found that the expression of SPAG5 also affected the patients’ response to immunotherapy based on the GSE61676 dataset. Therefore, we speculated that SPAG5 may affect immune regulation and immunotherapy in patients with EC.

DNA CNVs are the most common genetic alterations that affect cancer development and progression as they regulate the expression of cancer-associated genes^70–72^. Our study found that the copy number of SPAG5 was increased in EC. We speculate that altered SPAG5 expression and SPAG5 dysfunction in EC may be related to alterations in the chromosomal structure. SPAG5 abnormalities may cause abnormal changes in cell cycle, apoptosis, and immune response^48–50^. Moreover, our in vitro studies revealed that SPAG5 can regulate the migration, invasion, apoptosis and cell cycle of EC cells, which is consistent with the results of the functional analyses. These results further demonstrated that SPAG5 may play an important regulatory role in the progression of EC by regulating the migration, apoptosis, and cell cycle of cancer cells.

However, this study had some limitations. The clinical sample size in qRT-PCR validation was too small. To further explain the expression level of SPAG5 in EC, a large number of clinical samples will be collected for study at a later stage. It is not clear whether the identified SPAG5 related genes and miRNAs are involved in the mechanism of SPAG5 regulating EC, and further research is needed. The molecular mechanism of the impact of SPAG5 on the immune microenvironment of EC patients is still unclear and needs to be further explored. In addition, animal model validation was also lacking in this study. In short, the specific molecular regulatory mechanism of SPAG5 in EC is still unclear, and a large number of in vivo and in vitro studies are needed to investigate its role in EC occurrence and progression.

This study showed that SPAG5 was highly expressed and mutated in EC. Patients with EC and high expression of SPAG5 have a short survival time. GeneMANIA and STRING databases analyses showed the highest interaction relationship between SPAG5 and KNSTRN (a hub gene of EC). Functional enrichment analysis revealed that SPAG5 may be involved in a variety of biological pathways, which requires further study. SPAG5 also affected the level of immune cell infiltration in the TIME and the expression of immune checkpoints LAG3 and TIGIT in patients with EC and may be involved in the immunotherapy response of these patients. In addition, SPAG5 can regulate the migration, invasion, apoptosis, and cell cycle of EC cells, laying the foundation for the diagnosis and treatment of EC. In short, the results of this study lay the foundation for further understanding the molecular mechanisms of EC involving SPAG5 and contribute to the diagnosis and clinical management of patients with EC. Further study and elucidation of SPAG5 in EC will lead to new options for patient treatment.

### Supplementary Information

Supplementary Information 1.

Supplementary Figures.

Supplementary Information 3.

Supplementary Table S1.

Supplementary Table S2.

Supplementary Table S3.

Supplementary Table S4.

Supplementary Table S5.

Supplementary Table S6.

## Data Availability

The data used and analyzed during the current study are available from public databases. Corresponding data can be obtained by searching for the relevant disease name or gene name in the public databases used in this study. The databases used in the current study are UALCAN (http://ualcan.path.uab.edu/analysis.html), cBioPortal (http://cbioportal.org), OncoLnc (www.oncolnc.org/), GEPIA (http://gepia.cancer-pku.cn/), LinkedOmics (http://www.linkedomics.org/login.php). The relevant EC data was downloaded on 23 July 2020.
