# Supplementary material for: Upregulation of sperm-associated antigen 5 expression in endometrial carcinoma was associated with poor prognosis and immune dysregulation, and promoted cell migration and invasion
Source: Sci Rep. 2024 Jun 11;14:13415. doi: 10.1038/s41598-024-64354-4 (PMC11166665; doi:10.1038/s41598-024-64354-4)

**qRT-PCR amplification curve and product dissolution curve**

Tissue sample

GAPDH (Internal reference)

**
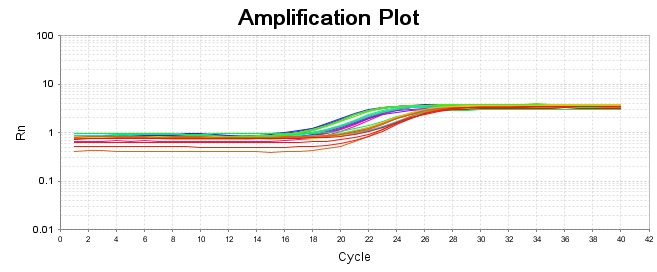

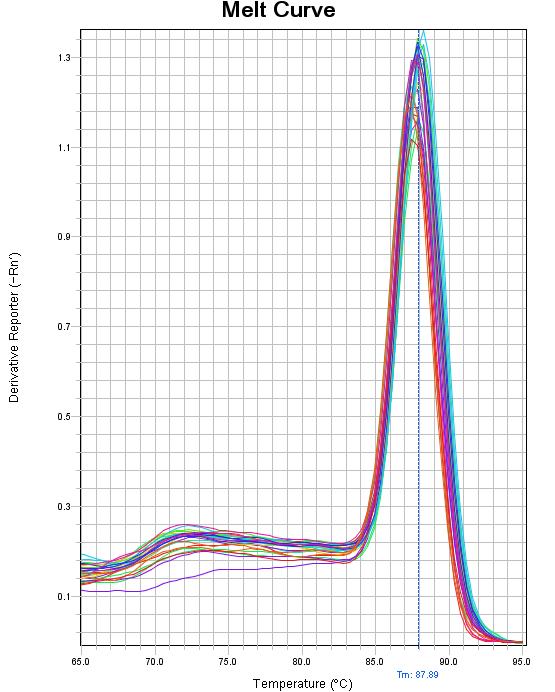
**

ACTB (Internal reference)

**
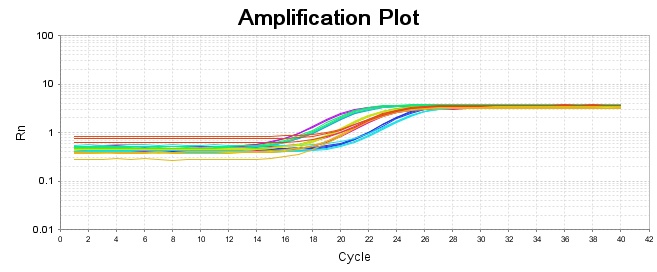

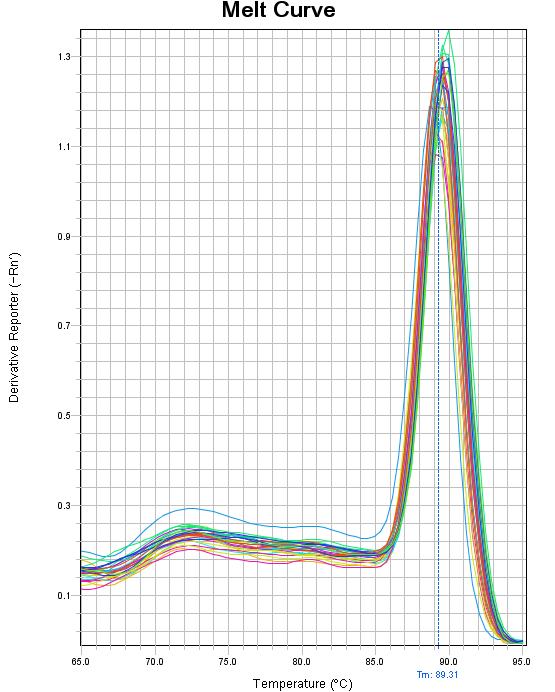
**

SPAG5

**
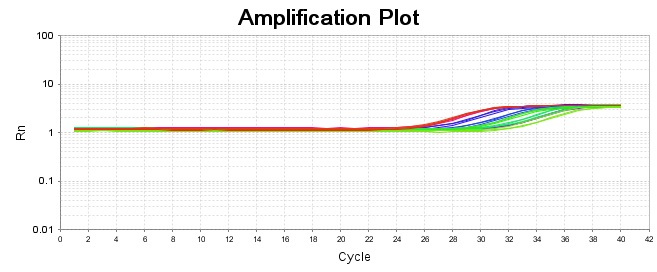

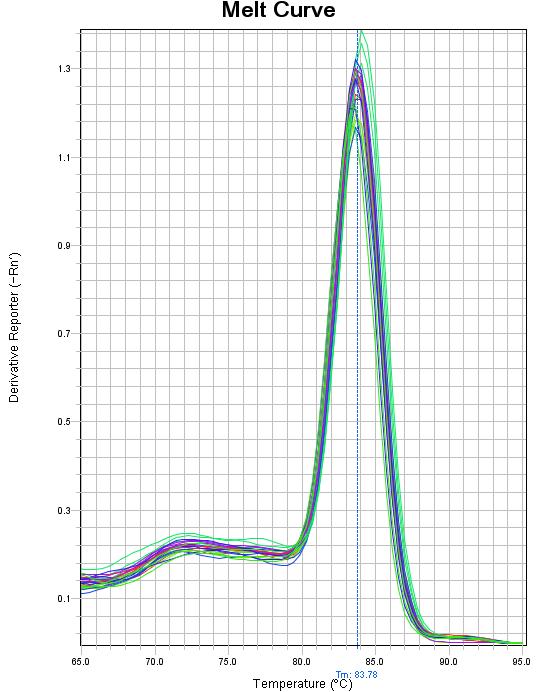
**

Cell sample

ACTB (Internal reference)


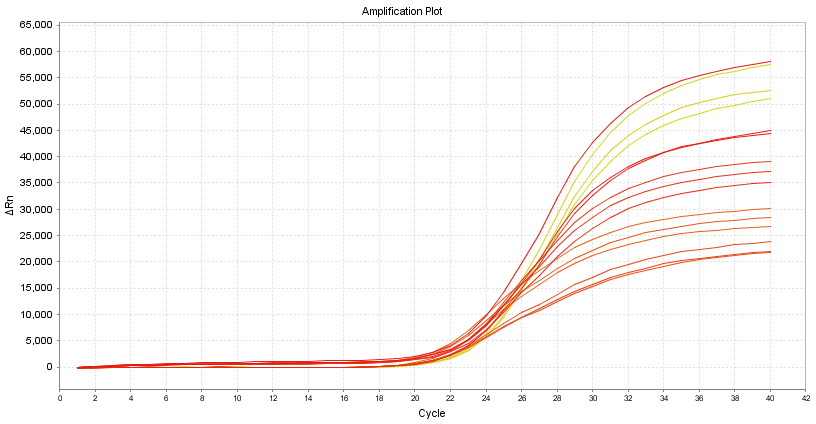

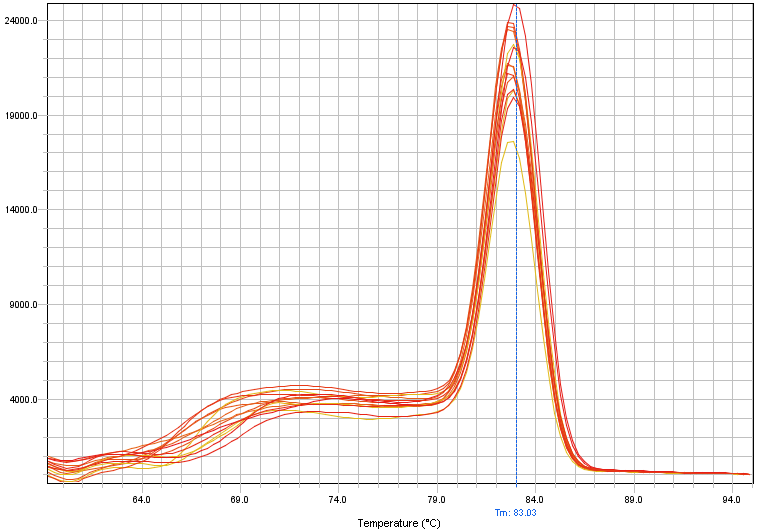


SPAG5


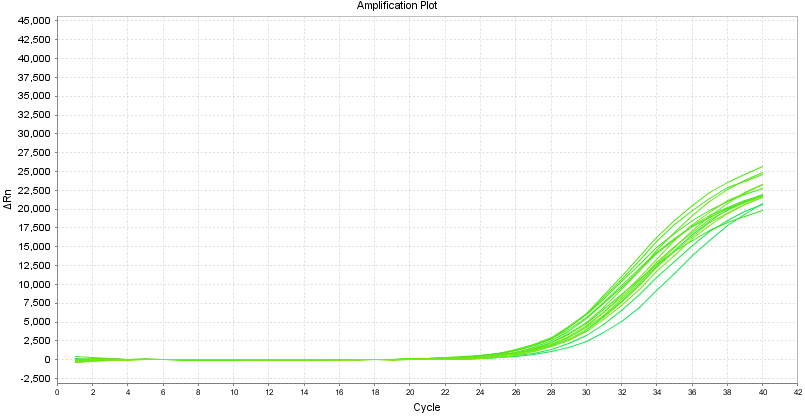

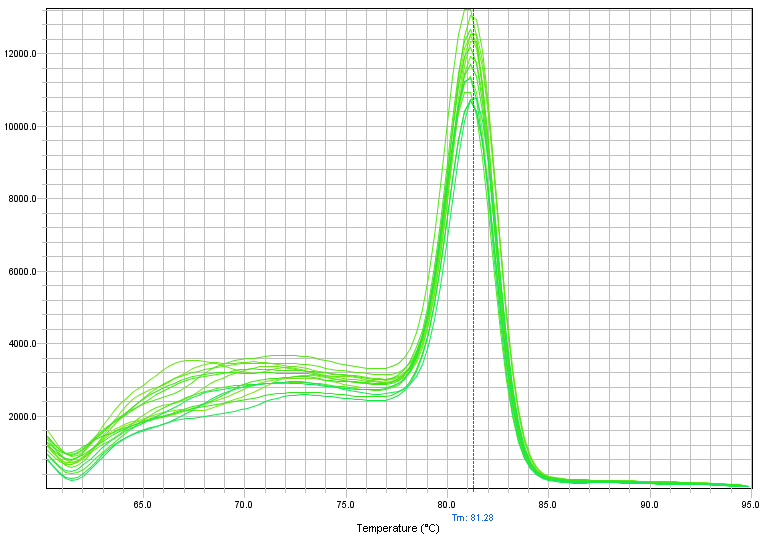


Cell sample—overexpression

ACTB (Internal reference)


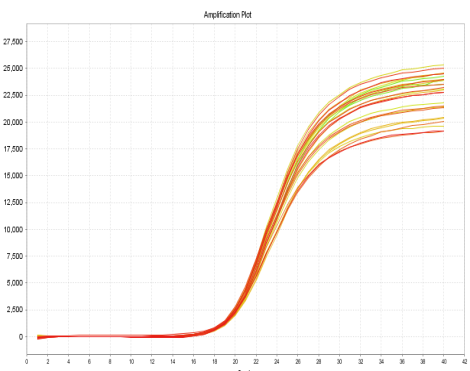

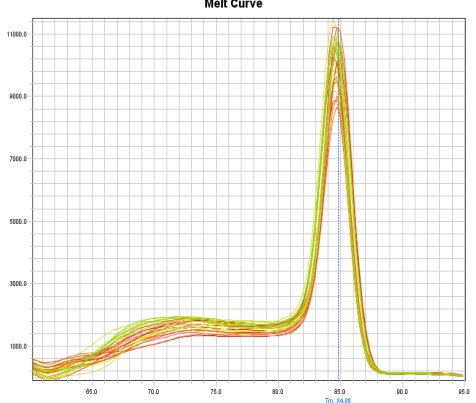


SPAG5


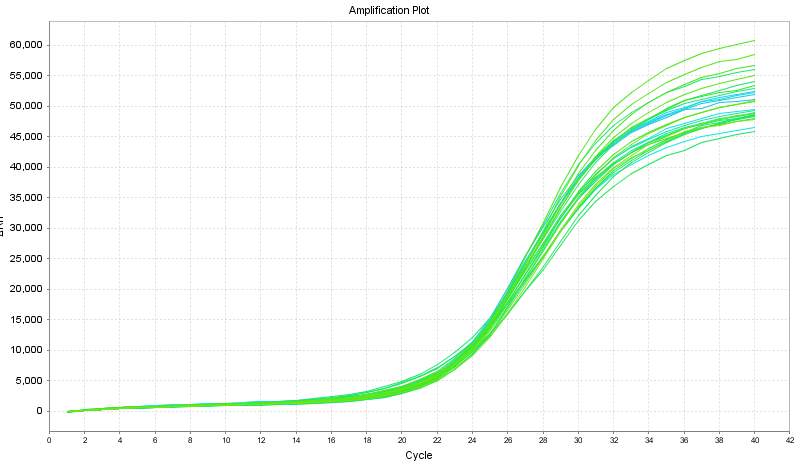

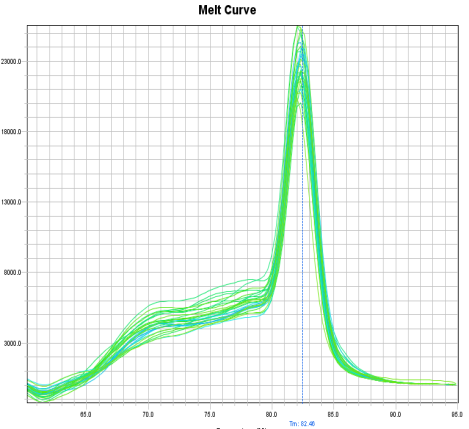


Cell sample—knockdown

ACTB (Internal reference)


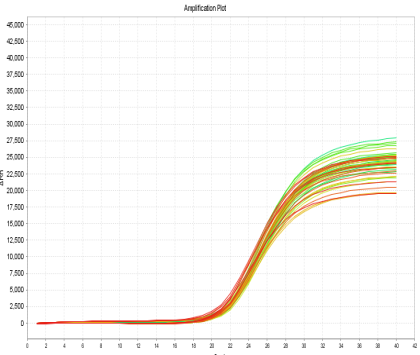

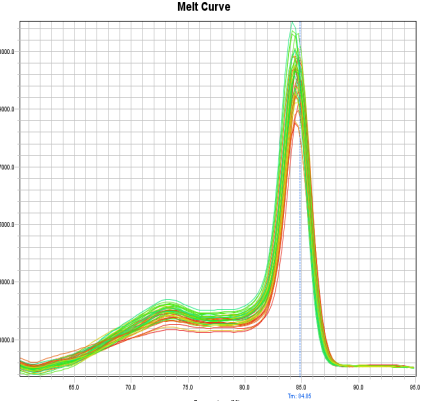


SPAG5


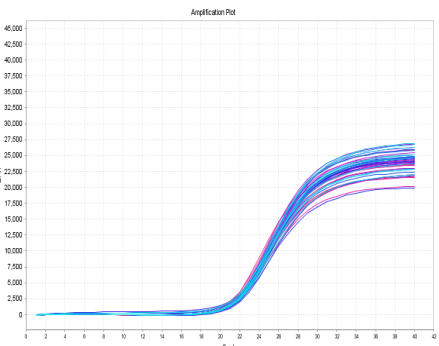

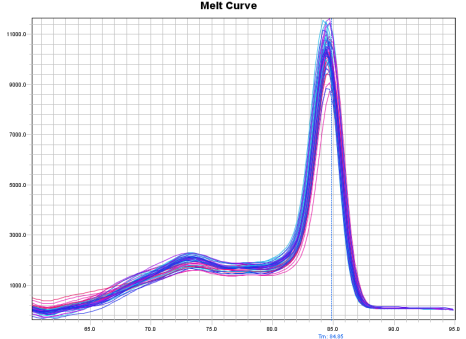

Supplement: Supplementary file 1 — Supplementary Information 1. [file 41598_2024_64354_MOESM1_ESM.docx]
