# Supplementary material for: Upregulation of sperm-associated antigen 5 expression in endometrial carcinoma was associated with poor prognosis and immune dysregulation, and promoted cell migration and invasion
Source: Sci Rep. 2024 Jun 11;14:13415. doi: 10.1038/s41598-024-64354-4 (PMC11166665; doi:10.1038/s41598-024-64354-4)
Supplement: Supplementary file 2 — Supplementary Figures. [file 41598_2024_64354_MOESM2_ESM.docx]

**Supplementary Figure**


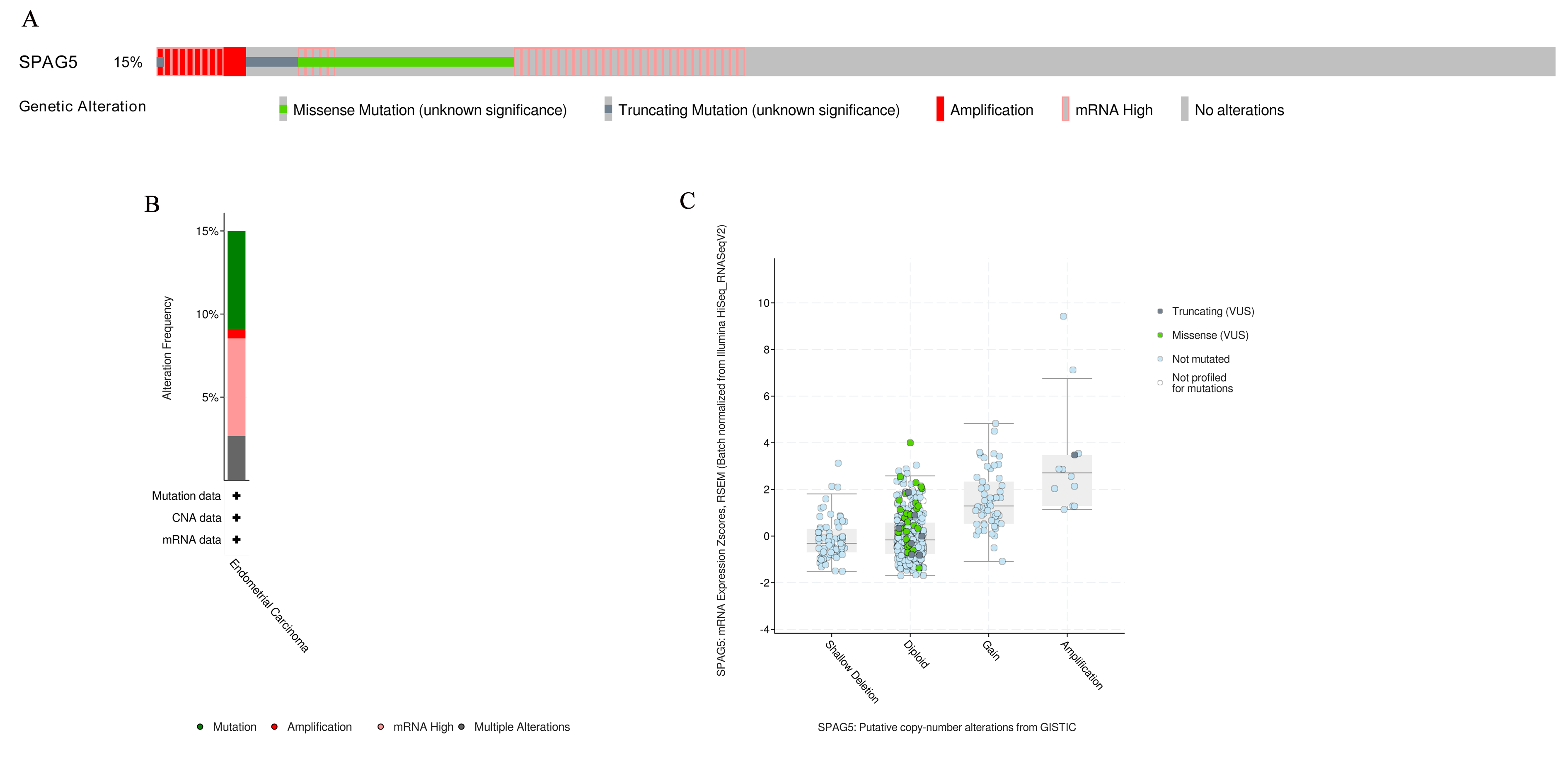


**Figure S1.** Frequency and type of SPAG5 alterations in EC.

**A,** SPAG5 alteration in EC; **B,** Summary of SPAG5 alteration in EC; **C,** Expression of SPAG5 in different altered states. EC, endometrial carcinoma.


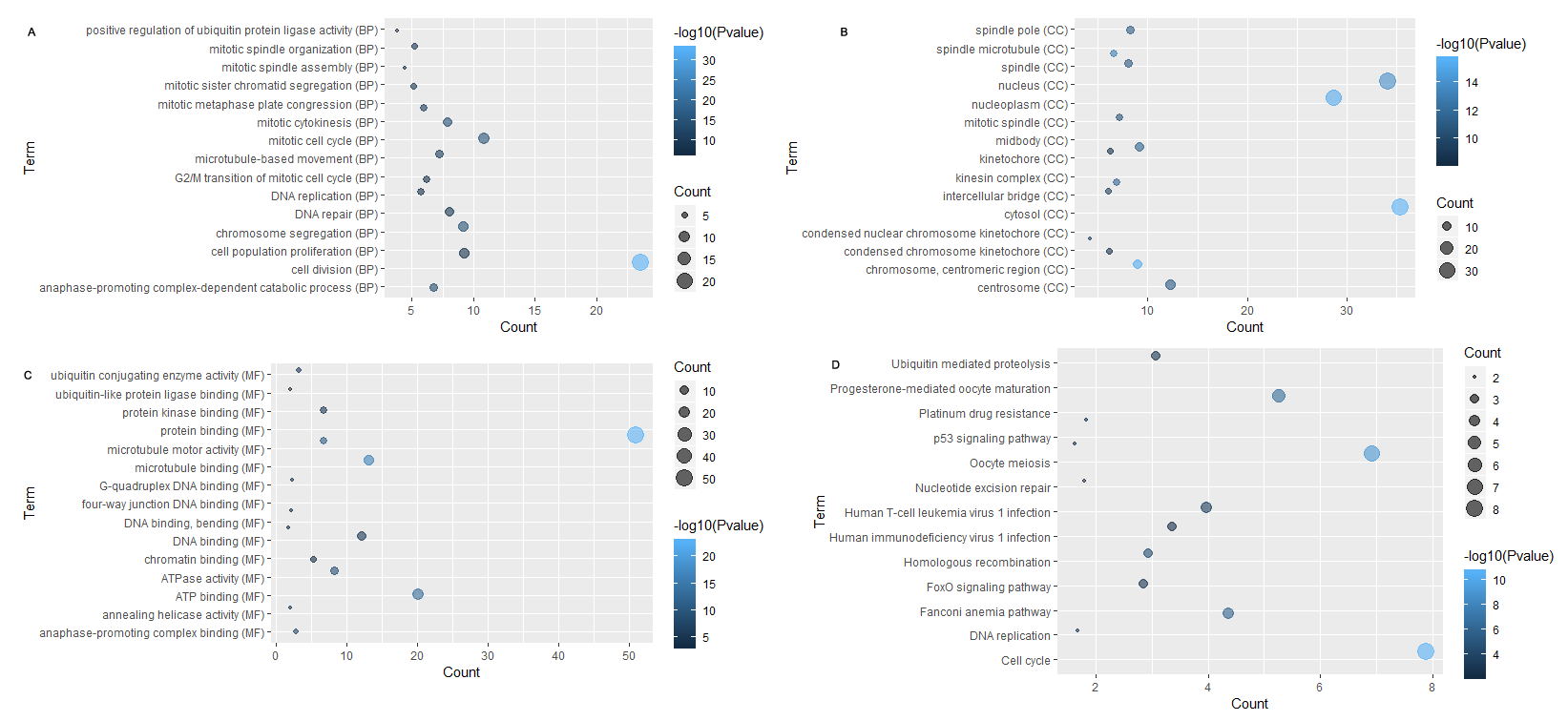


**Figure S2.** Functional enrichment of genes similar to SPAG5.

**A,** GO-enriched BP terms (top 15) of genes similar to SPAG5; **B,** GO-enriched CC terms (top 15) of genes similar to SPAG5; **C,** GO-enriched CC terms (top 15) of genes similar to SPAG5; **D,** KEGG enrichment of genes similar to SPAG5.


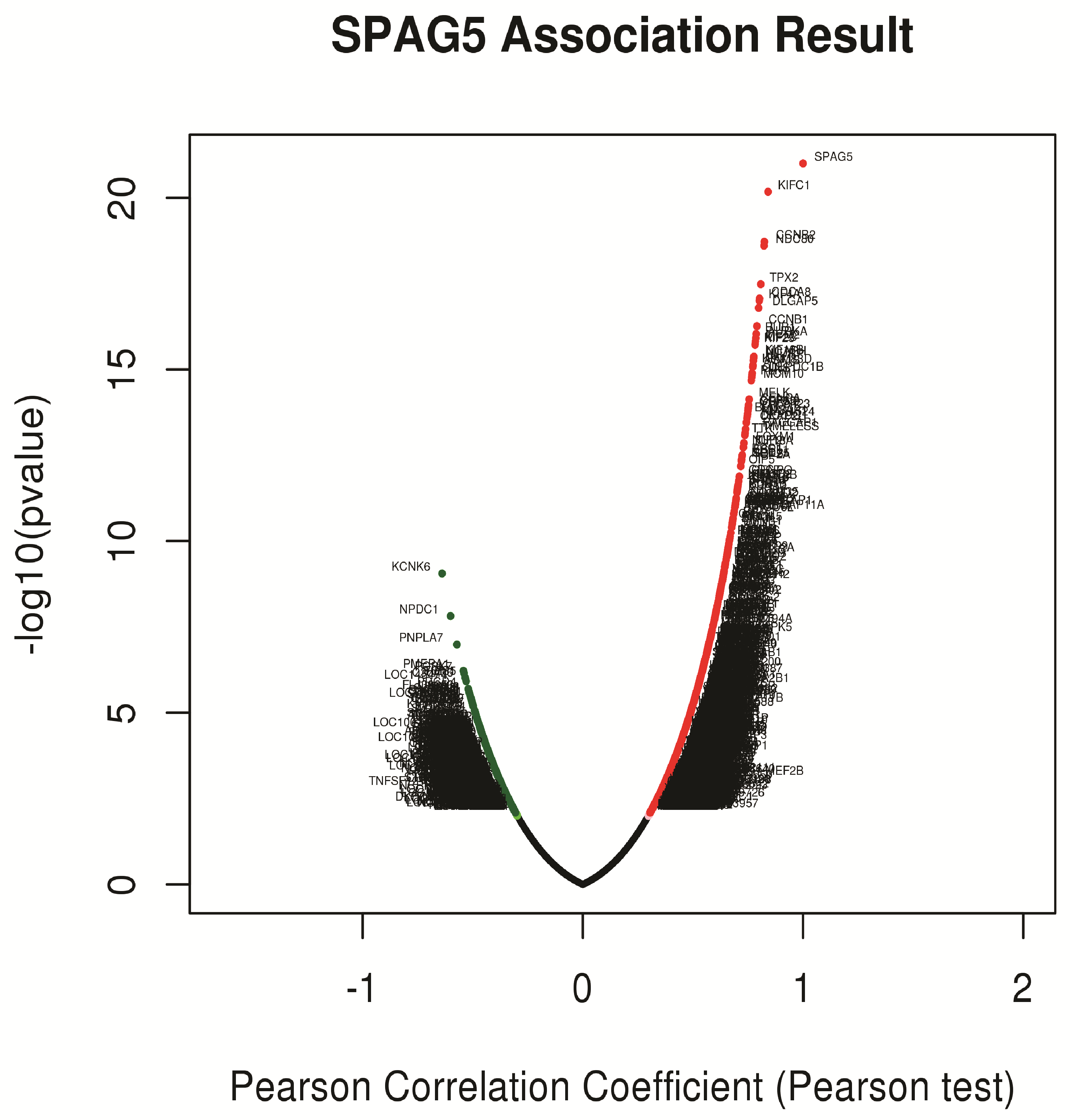


**Figure S3.** Volcano plot of genes positively and negatively correlated with SPAG5 expression;


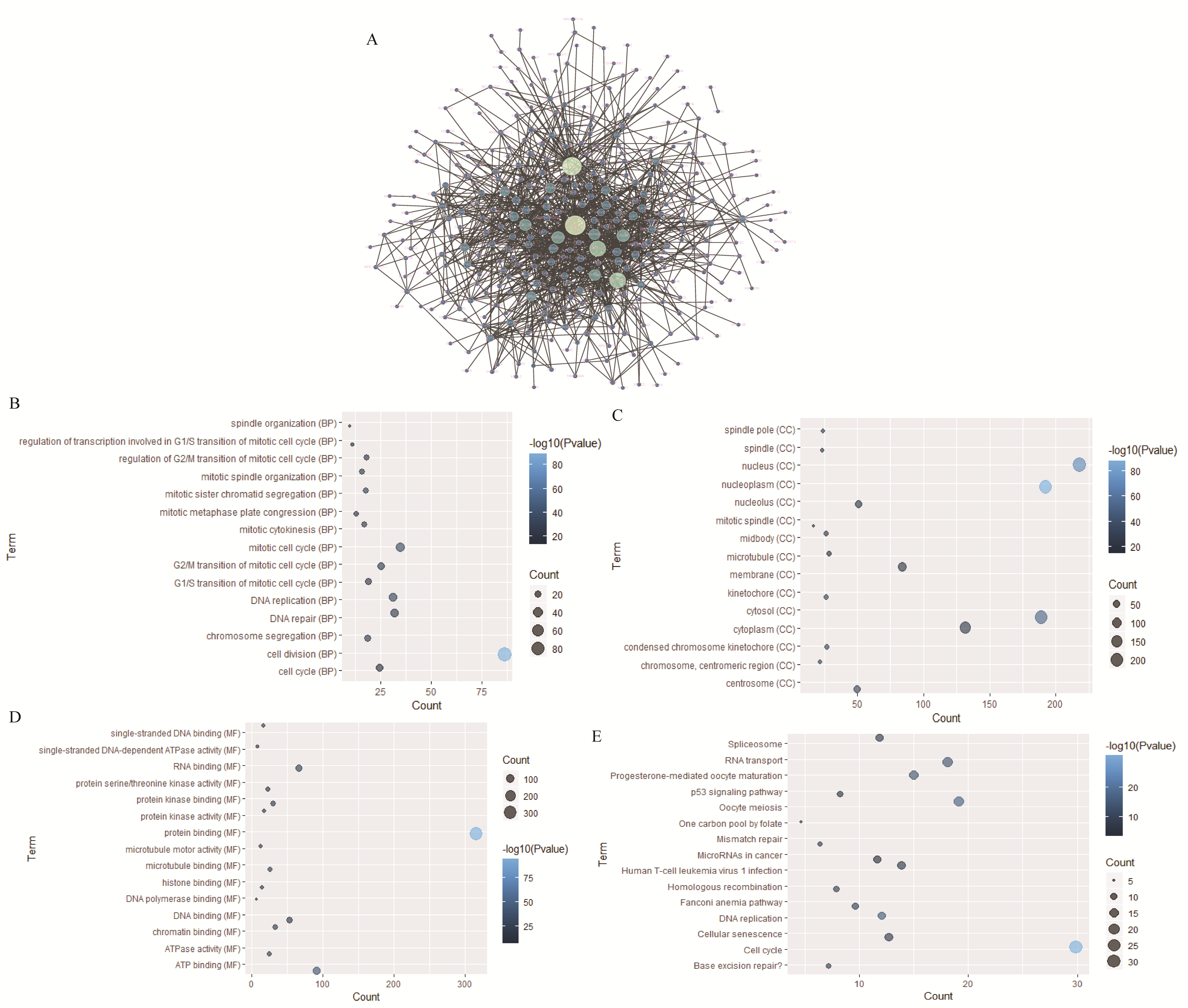


**Figure S4.** Interaction and functional enrichment of SPAG5 co-expressed genes.

**A,** Protein interaction network of SPAG5 co-expressed genes. The larger the Degree, the larger the node and the lighter the node color. **B,** GO-enriched BP terms (top 15) of SPAG5 co-expressed genes; **C,** GO-enriched CC terms (top 15) of SPAG5 co-expressed genes; **D,** GO-enriched MF terms (top 15) of SPAG5 co-expressed genes; **E,** KEGG enrichment (top 15) of SPAG5 co-expressed genes.


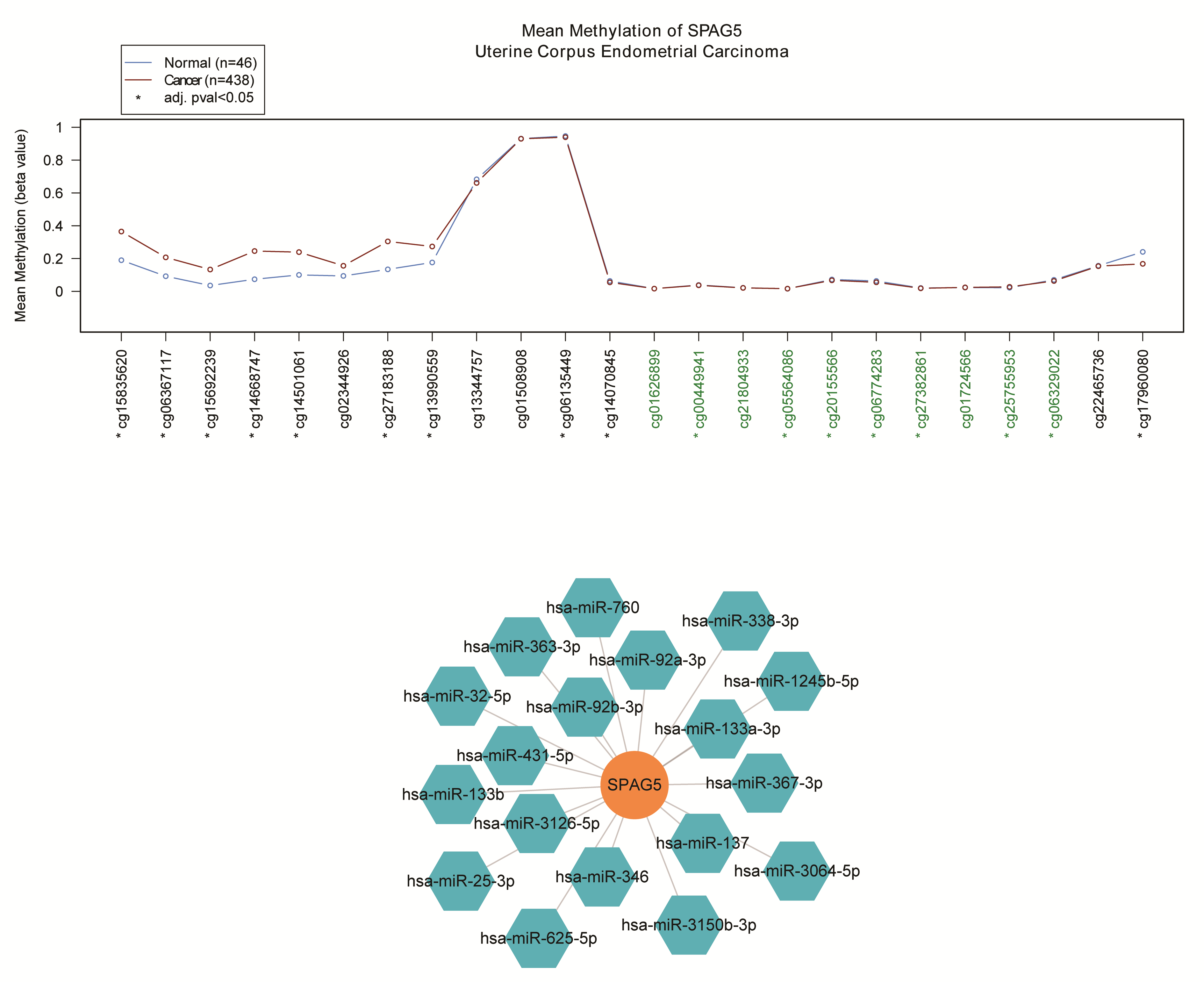


**Figure S5.** Identification of SPAG5 methylation sites and construction of SPAG5-miRNA network.

**A,** Identification of SPAG5 methylation sites; **B,** Construction of SPAG5-miRNA network.
