# Supplementary figures and images for: Upregulation of sperm-associated antigen 5 expression in endometrial carcinoma was associated with poor prognosis and immune dysregulation, and promoted cell migration and invasion
Source: Sci Rep. 2024 Jun 11;14:13415. doi: 10.1038/s41598-024-64354-4 (PMC11166665; doi:10.1038/s41598-024-64354-4)

Actin full length uncropped blots


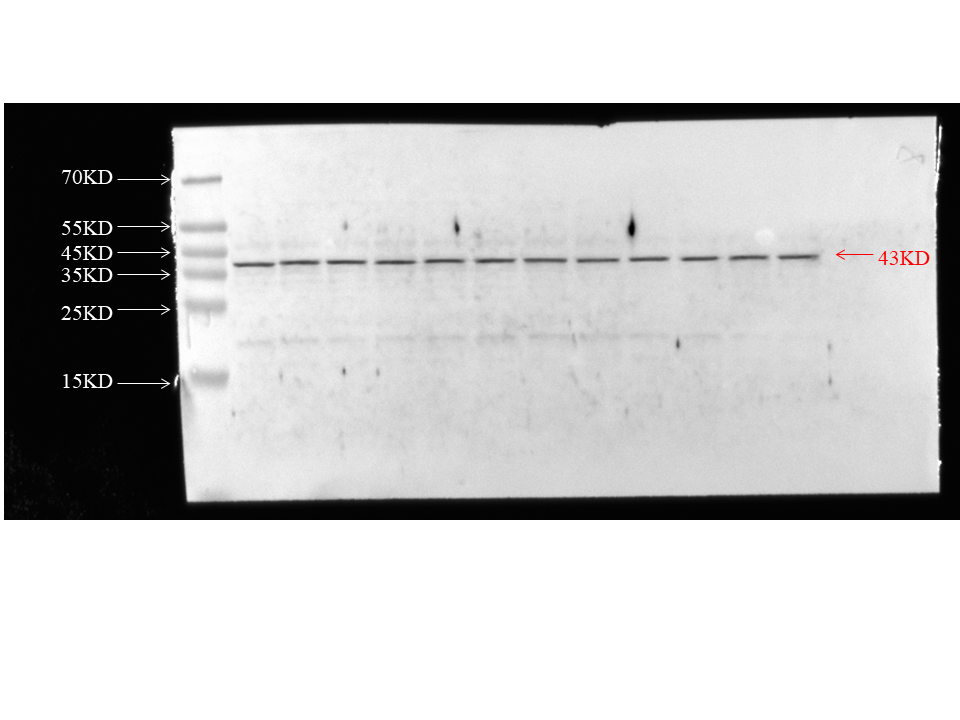

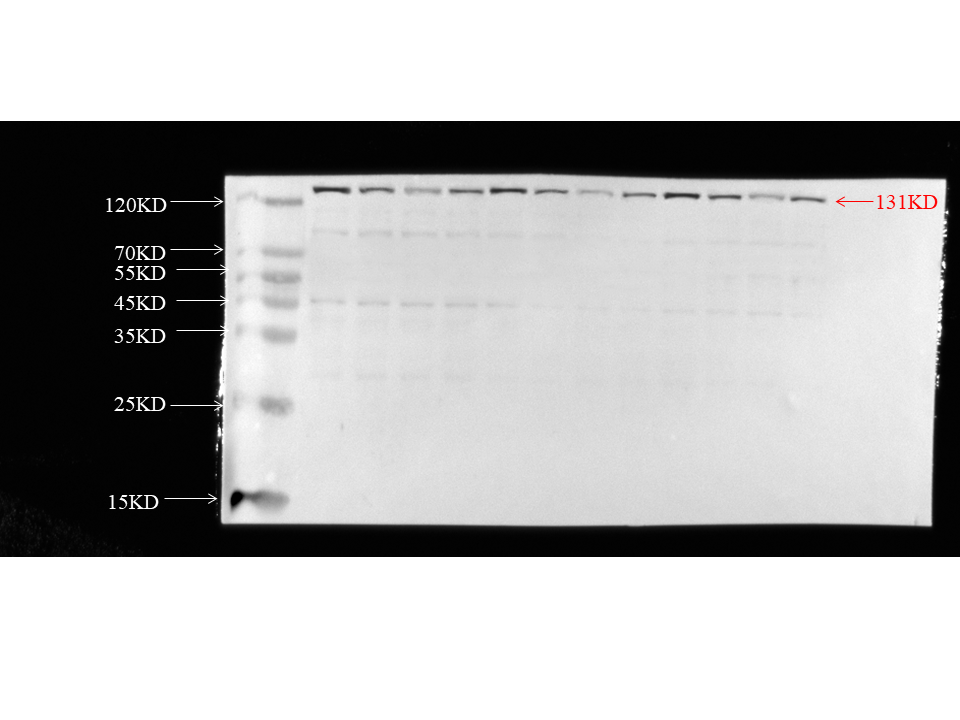


SPAG5 full length uncropped blots

Supplement: Supplementary file 3 — Supplementary Information 3. [file 41598_2024_64354_MOESM3_ESM.docx]
