# Supplementary material for: Upregulation of sperm-associated antigen 5 expression in endometrial carcinoma was associated with poor prognosis and immune dysregulation, and promoted cell migration and invasion
Source: Sci Rep. 2024 Jun 11;14:13415. doi: 10.1038/s41598-024-64354-4 (PMC11166665; doi:10.1038/s41598-024-64354-4)
Supplement: Supplementary file 4 — Supplementary Table S1. [file 41598_2024_64354_MOESM4_ESM.docx]

**Table S1**. Statistical significance of SPAG5 expression

| **Comparison** | **Statistical significance (p-value)** | **Adjustment p-value (false discovery rate, FDR)** |
| --- | --- | --- |
| Normal-vs-Primary cancer | 1.62458935193399E-12 | 4.54885018541517E-11 |
| Normal-vs-Age(21-40Yrs) | 7.73880000000116E-05 | 0.002166864 |
| Normal-vs-Age(41-60Yrs) | <1E-12 | <2.8E-11 |
| Normal-vs-Age(61-80Yrs) | 1.62447832963153E-12 | 4.54853932296828E-11 |
| Normal-vs-Age(81-100Yrs) | 5.82911496849192E-12 | 1.63215219117774E-10 |
| Normal-vs-Endometrioid | 1.62447832963153E-12 | 4.54853932296828E-11 |
| Normal-vs-Serous | <1E-12 | <2.8E-11 |
| Normal-vs-Mixed serous and endometrioid | 1.20309999962487E-07 | 3.36867999894964E-06 |
| Endometrioid -vs-Serous | 3.34360000020517E-07 | 9.36208000057447E-06 |
| Normal-vs-Pre-Menopause | 2.1273000028188E-09 | 5.95644000789264E-08 |
| Normal-vs-Peri-Menopause | 3.50239999999946E-05 | 0.000980672 |
| Normal-vs-Post-Menopause | <1E-12 | <2.8E-11 |
| Normal-vs-Normal_Weight | <1E-12 | <2.8E-11 |
| Normal-vs-Extreme_Weight | <1E-12 | <2.8E-11 |
| Normal-vs-Obese | <1E-12 | <2.8E-11 |
| Normal-vs-Extreme_Obese | <1E-12 | <2.8E-11 |
| Normal_Weight-vs-Extreme_Weight | 1.763960E-02 | 0.4939088 |
| Normal_Weight-vs-Obese | 2.321800E-02 | 0.650104 |
| Normal_Weight-vs-Extreme_Obese | 7.352200E-04 | 0.02058616 |
| Normal-vs-Caucasian | 1.62458935193399E-12 | 4.54885018541517E-11 |
| Normal-vs-African American | 1.62447832963153E-12 | 4.54853932296828E-11 |
| Normal-vs-Asian | 1.9940899999904E-05 | 0.000558345 |
| Normal-vs-Stage1 | <1E-12 | <2.8E-11 |
| Normal-vs-Stage2 | 1.62458935193399E-12 | 4.54885018541517E-11 |
| Normal-vs-Stage3 | <1E-12 | <2.8E-11 |
| Normal-vs-Stage4 | 3.60910001884207E-09 | 1.01054800527578E-07 |
| Stage1-vs-Stage3 | 7.221300E-04 | 0.02021964 |
| Stage1-vs-Stage4 | 2.924000E-02 | 0.81872 |
