# Supplementary material for: Upregulation of sperm-associated antigen 5 expression in endometrial carcinoma was associated with poor prognosis and immune dysregulation, and promoted cell migration and invasion
Source: Sci Rep. 2024 Jun 11;14:13415. doi: 10.1038/s41598-024-64354-4 (PMC11166665; doi:10.1038/s41598-024-64354-4)
Supplement: Supplementary file 5 — Supplementary Table S2. [file 41598_2024_64354_MOESM5_ESM.docx]

**Table S2 Interaction score between interacting genes based on STRING database**

| **Node1** | **Node2** | **Node1_string_id** | **Node2_string_id** | **Combined_score** |
| --- | --- | --- | --- | --- |
| ATAD3A | ATAD3B | 9606.ENSP00000368030 | 9606.ENSP00000500094 | 0.64 |
| AURKA | KNSTRN | 9606.ENSP00000216911 | 9606.ENSP00000249776 | 0.541 |
| AURKA | TROAP | 9606.ENSP00000216911 | 9606.ENSP00000257909 | 0.563 |
| AURKA | SPAG5 | 9606.ENSP00000216911 | 9606.ENSP00000323300 | 0.8 |
| AURKA | CDC25C | 9606.ENSP00000216911 | 9606.ENSP00000321656 | 0.831 |
| AURKA | CENPF | 9606.ENSP00000216911 | 9606.ENSP00000355922 | 0.902 |
| AURKA | CDK1 | 9606.ENSP00000216911 | 9606.ENSP00000378699 | 0.951 |
| AURKA | CCNB2 | 9606.ENSP00000216911 | 9606.ENSP00000288207 | 0.988 |
| CCNB2 | KNSTRN | 9606.ENSP00000288207 | 9606.ENSP00000249776 | 0.437 |
| CCNB2 | TROAP | 9606.ENSP00000288207 | 9606.ENSP00000257909 | 0.674 |
| CCNB2 | SPAG5 | 9606.ENSP00000288207 | 9606.ENSP00000323300 | 0.76 |
| CCNB2 | CENPF | 9606.ENSP00000288207 | 9606.ENSP00000355922 | 0.974 |
| CCNB2 | CDC25C | 9606.ENSP00000288207 | 9606.ENSP00000321656 | 0.993 |
| CCNB2 | CDK1 | 9606.ENSP00000288207 | 9606.ENSP00000378699 | 0.999 |
| CDC25C | TROAP | 9606.ENSP00000321656 | 9606.ENSP00000257909 | 0.657 |
| CDC25C | PRKDC | 9606.ENSP00000321656 | 9606.ENSP00000313420 | 0.643 |
| CDC25C | SPAG5 | 9606.ENSP00000321656 | 9606.ENSP00000323300 | 0.602 |
| CDC25C | CENPF | 9606.ENSP00000321656 | 9606.ENSP00000355922 | 0.618 |
| CDC25C | CDK1 | 9606.ENSP00000321656 | 9606.ENSP00000378699 | 0.999 |
| CDK1 | KNSTRN | 9606.ENSP00000378699 | 9606.ENSP00000249776 | 0.641 |
| CDK1 | TROAP | 9606.ENSP00000378699 | 9606.ENSP00000257909 | 0.547 |
| CDK1 | PRKDC | 9606.ENSP00000378699 | 9606.ENSP00000313420 | 0.537 |
| CDK1 | SPAG5 | 9606.ENSP00000378699 | 9606.ENSP00000323300 | 0.927 |
| CDK1 | CENPF | 9606.ENSP00000378699 | 9606.ENSP00000355922 | 0.978 |
| CDK1 | DCLRE1B | 9606.ENSP00000378699 | 9606.ENSP00000498042 | 0.427 |
| CDK5RAP2 | CEP72 | 9606.ENSP00000343818 | 9606.ENSP00000264935 | 0.419 |
| CDK5RAP2 | SPAG5 | 9606.ENSP00000343818 | 9606.ENSP00000323300 | 0.483 |
| CDK5RAP2 | RPGRIP1L | 9606.ENSP00000343818 | 9606.ENSP00000493946 | 0.628 |
| CEBPD | MED1 | 9606.ENSP00000386165 | 9606.ENSP00000300651 | 0.546 |
| CEBPD | PRKDC | 9606.ENSP00000386165 | 9606.ENSP00000313420 | 0.436 |
| CENPF | KNSTRN | 9606.ENSP00000355922 | 9606.ENSP00000249776 | 0.623 |
| CENPF | TROAP | 9606.ENSP00000355922 | 9606.ENSP00000257909 | 0.764 |
| CENPF | SPAG5 | 9606.ENSP00000355922 | 9606.ENSP00000323300 | 0.905 |
| CEP72 | SPAG5 | 9606.ENSP00000264935 | 9606.ENSP00000323300 | 0.486 |
| DCAF7 | TROAP | 9606.ENSP00000483236 | 9606.ENSP00000257909 | 0.518 |
| DCLRE1B | PRKDC | 9606.ENSP00000498042 | 9606.ENSP00000313420 | 0.798 |
| DCLRE1B | SPAG5 | 9606.ENSP00000498042 | 9606.ENSP00000323300 | 0.68 |
| KNSTRN | SPAG5 | 9606.ENSP00000249776 | 9606.ENSP00000323300 | 0.983 |
| MED1 | SPAG5 | 9606.ENSP00000300651 | 9606.ENSP00000323300 | 0.473 |
| SPAG5 | TROAP | 9606.ENSP00000323300 | 9606.ENSP00000257909 | 0.813 |
