# Supplementary material for: Upregulation of sperm-associated antigen 5 expression in endometrial carcinoma was associated with poor prognosis and immune dysregulation, and promoted cell migration and invasion
Source: Sci Rep. 2024 Jun 11;14:13415. doi: 10.1038/s41598-024-64354-4 (PMC11166665; doi:10.1038/s41598-024-64354-4)
Supplement: Supplementary file 7 — Supplementary Table S4. [file 41598_2024_64354_MOESM7_ESM.docx]

**Table S4 Gene similar to SPAG5 in endometrial carcinoma tissue**

| **Gene Symbol** | **Gene ID** | **PCC** |
| --- | --- | --- |
| TROAP | ENSG00000135451.12 | 0.65 |
| CCNB2 | ENSG00000157456.7 | 0.61 |
| KIF2C | ENSG00000142945.12 | 0.61 |
| PRC1 | ENSG00000198901.13 | 0.6 |
| UBE2C | ENSG00000175063.16 | 0.6 |
| PIF1 | ENSG00000140451.12 | 0.6 |
| TACC3 | ENSG00000013810.18 | 0.6 |
| BLM | ENSG00000197299.10 | 0.59 |
| BIRC5 | ENSG00000089685.14 | 0.59 |
| PTTG1 | ENSG00000164611.12 | 0.59 |
| CENPA | ENSG00000115163.14 | 0.58 |
| RP13-753N3.3 | ENSG00000275185.1 | 0.58 |
| HMGB2 | ENSG00000164104.11 | 0.57 |
| CDC20 | ENSG00000117399.13 | 0.57 |
| CCNB1 | ENSG00000134057.14 | 0.57 |
| TOP2A | ENSG00000131747.14 | 0.56 |
| CDCA3 | ENSG00000111665.11 | 0.56 |
| OIP5 | ENSG00000104147.8 | 0.56 |
| FANCG | ENSG00000221829.9 | 0.56 |
| PLK1 | ENSG00000166851.14 | 0.56 |
| SKA2 | ENSG00000182628.12 | 0.56 |
| HJURP | ENSG00000123485.11 | 0.56 |
| UBE2SP2 | ENSG00000224126.2 | 0.55 |
| BUB1B | ENSG00000156970.12 | 0.55 |
| KIFC1 | ENSG00000237649.7 | 0.55 |
| NCAPH | ENSG00000121152.9 | 0.55 |
| ARHGEF39 | ENSG00000137135.17 | 0.55 |
| HAUS8 | ENSG00000131351.14 | 0.54 |
| TK1 | ENSG00000167900.11 | 0.54 |
| MTFR2 | ENSG00000146410.11 | 0.54 |
| KIF22 | ENSG00000079616.12 | 0.54 |
| RP4-616B8.5 | ENSG00000274825.1 | 0.53 |
| SGOL1 | ENSG00000129810.14 | 0.53 |
| NDC80 | ENSG00000080986.12 | 0.52 |
| MELK | ENSG00000165304.7 | 0.52 |
| ATP5G1P4 | ENSG00000227440.1 | 0.52 |
| SKA1 | ENSG00000154839.9 | 0.52 |
| NUSAP1 | ENSG00000137804.12 | 0.52 |
| CDC25C | ENSG00000158402.18 | 0.52 |
| CDCA8 | ENSG00000134690.10 | 0.52 |
| FAM72B | ENSG00000188610.12 | 0.52 |
| CEP55 | ENSG00000138180.15 | 0.52 |
| EME1 | ENSG00000154920.14 | 0.52 |
| KIF4A | ENSG00000090889.11 | 0.52 |
| ECT2 | ENSG00000114346.13 | 0.52 |
| POLDIP2 | ENSG00000004142.11 | 0.51 |
| POLE2 | ENSG00000100479.12 | 0.51 |
| UBE2T | ENSG00000077152.9 | 0.51 |
| RFC4 | ENSG00000163918.10 | 0.51 |
| C16orf59 | ENSG00000162062.14 | 0.51 |
| RAD54L | ENSG00000085999.11 | 0.51 |
| KIF23 | ENSG00000137807.13 | 0.51 |
| KIF20A | ENSG00000112984.11 | 0.51 |
| H2AFV | ENSG00000105968.18 | 0.5 |
| CDC45 | ENSG00000093009.9 | 0.5 |
| ASF1B | ENSG00000105011.8 | 0.5 |
| TPX2 | ENSG00000088325.15 | 0.5 |
| NASP | ENSG00000132780.16 | 0.5 |
| KIF15 | ENSG00000163808.16 | 0.5 |
| NUF2 | ENSG00000143228.12 | 0.5 |
| UBE2S | ENSG00000108106.13 | 0.5 |
| HIRIP3 | ENSG00000149929.15 | 0.5 |
