# Supplementary material for: Upregulation of sperm-associated antigen 5 expression in endometrial carcinoma was associated with poor prognosis and immune dysregulation, and promoted cell migration and invasion
Source: Sci Rep. 2024 Jun 11;14:13415. doi: 10.1038/s41598-024-64354-4 (PMC11166665; doi:10.1038/s41598-024-64354-4)
Supplement: Supplementary file 8 — Supplementary Table S5. [file 41598_2024_64354_MOESM8_ESM.docx]

**Table S5 Clinical information of patient samples used for qRT-PCR analysis**

| **Number** | **Age** | **Body Mass Index (BMI)** | **Blood pressure** | **Period** | **Estrogen stimulation (Yes or No)** | **Previous history of endometrial adenocarcinoma** | **Family history** | **Pathological examination results** | **Stages** | **Primary tumor** | **Lymphatic or distal metastases** | **Treatment method** |
| --- | --- | --- | --- | --- | --- | --- | --- | --- | --- | --- | --- | --- |
| 1 | 73 | 18.88 | 125/69 | Post-menopause | No | No | No | Endometrial malignancy | Stage IIIB | Yes | No | Surgery+late chemotherapy and radiotherapy |
| 2 | 66 | 23.88 | 96/65 | Post-menopause | No | No | No | Endometrioid adenocarcinoma | Stage IB | Yes | No | Surgery+late chemotherapy and radiotherapy |
| 3 | 65 | 23.4 | 146/97 | Post-menopause | No | No | No | Endometrioid adenocarcinoma | Stage IA | Yes | No | Surgery |
| 4 | 52 | 22.64 | 138/81 | Post-menopause | No | No | No | Endometrioid adenocarcinoma | Stage IA | Yes | No | Surgery |
| 5 | 55 | 24.12 | 148/106 | Post-menopause | No | No | No | Endometrioid adenocarcinoma | Stage IA | Yes | No | Surgery |
| 6 | 50 | 25.9 | 171/92 | Post-menopause | No | No | No | Endometrioid adenocarcinoma | Stage IA | Yes | No | Surgery |
| 7 | 53 | 25.5 | 153/96 | Post-menopause | No | No | No | Endometrioid adenocarcinoma | Stage IA | Yes | No | Surgery |
| 8 | 72 | 23.3 | 147/91 | Post-menopause | No | No | No | Endometrioid adenocarcinoma | Stage IIIB | Yes | No | Surgery+late chemotherapy and radiotherapy |
| 9 | 58 | 26.5 | 116/84 | Post-menopause | No | No | No | Endometrioid adenocarcinoma | Stage IB | Yes | No | Surgery+late chemotherapy and radiotherapy |
