# Supplementary material for: Upregulation of sperm-associated antigen 5 expression in endometrial carcinoma was associated with poor prognosis and immune dysregulation, and promoted cell migration and invasion
Source: Sci Rep. 2024 Jun 11;14:13415. doi: 10.1038/s41598-024-64354-4 (PMC11166665; doi:10.1038/s41598-024-64354-4)
Supplement: Supplementary file 9 — Supplementary Table S6. [file 41598_2024_64354_MOESM9_ESM.docx]

**Table S6 Primer sequences used for the quantitative real-time PCR (qRT-PCR)**

|  | **Primer name** | **Primer sequence (5’ to 3’)** |
| --- | --- | --- |
| Clinical | GAPDH-F (internal reference) | 5’-GGAGCGAGATCCCTCCAAAAT-3’ |
|  | GAPDH-R (internal reference) | 5’-GGCTGTTGTCATACTTCTCATGG-3’ |
|  | ACTB-F (internal reference) | 5’-CATGTACGTTGCTATCCAGGC-3’ |
|  | ACTB-R (internal reference) | 5’-CTCCTTAATGTCACGCACGAT-3’ |
|  | SPAG5-F | 5’-CTGAGCAGTAGAACTGAGGCT-3’ |
|  | SPAG5-R | 5’-TCCACATGATTGACACGGAAAT-3’ |
| Cell | SPAG5-F | 5’-GCTGGAAAATCTCCGCCAAA-3’ |
|  | SPAG5-R | 5’-GAAGGAGTAAACCAAGTCCCC-3’ |
|  | β-actin-F (internal reference) | 5’-ACTCTTCCAGCCTTCCTTCC-3’ |
|  | β-actin-R (internal reference) | 5’-CAATGCCAGGGTACATGGTG-3’ |

Cell: qRT-PCR was used for detection of SPAG5 expression level in cells.

Clinical: qRT-PCR was used for detection of SPAG5 expression level in clinical EC patients.
